# Differential effects of macrophage subtypes on SARS-CoV-2 infection in a human pluripotent stem cell-derived model

**DOI:** 10.1038/s41467-022-29731-5

**Published:** 2022-04-19

**Authors:** Qizhou Lian, Kui Zhang, Zhao Zhang, Fuyu Duan, Liyan Guo, Weiren Luo, Bobo Wing-Yee Mok, Abhimanyu Thakur, Xiaoshan Ke, Pedram Motallebnejad, Vlad Nicolaescu, Jonathan Chen, Chui Yan Ma, Xiaoya Zhou, Shuo Han, Teng Han, Wei Zhang, Adrian Y. Tan, Tuo Zhang, Xing Wang, Dong Xu, Jenny Xiang, Aimin Xu, Can Liao, Fang-Ping Huang, Ya-Wen Chen, Jie Na, Glenn Randall, Hung-fat Tse, Zhiwei Chen, Yin Chen, Huanhuan Joyce Chen

**Affiliations:** 1 Cord Blood Bank Center, Cord Blood Bank, Guangzhou Institute of Eugenics and Perinatology, Guangzhou Women and Children’s Medical Center, Guangzhou Medical University, Guangzhou, China; 2 HKUMed Laboratory of Cellular Therapeutics, and Department of Medicine, the University of Hong Kong, Hong Kong SAR, China; 3 The Pritzker School of Molecular Engineering, the University of Chicago, Chicago, IL 60637 USA; 4 The Ben May Department for Cancer Research, the University of Chicago, Chicago, IL 60637 USA; 5 Department of Pathology, The Second Affiliated Hospital of Southern University of Science and Technology, Shenzhen Third People’s Hospital, National Clinical Research Centre for Infectious Diseases, Shenzhen, China; 6 Department of Microbiology and State Key Laboratory for Emerging Infectious Diseases, Li Ka Shing Faculty of Medicine, The University of Hong Kong, Hong Kong SAR, China; 7 Microbiology, Biosciences Division, the University of Chicago, Chicago, IL 60637 USA; 8 McCormick School of Engineering, Northwestern University, Chicago, IL USA; 9 School of Biomedical Sciences, Li Ka Shing Faculty of Medicine, The University of Hong Kong, Hong Kong SAR, China; 10 Department of Medicine, Sandra and Edward Meyer Cancer Center, Weill Cornell Medicine, New York, NY 10021 USA; 11 Genomic Resource Core Facility, Weill Cornell Medicine, New York, NY 10065 USA; 12 State Key Laboratory of Pharmaceutical Biotechnology, Li Ka Shing Faculty of Medicine, The University of Hong Kong, Hong Kong SAR, China; 13 Institute for Advanced Study (IAS), Shenzhen University, Shenzhen, China; 14 Department of Otolaryngology, Icahn School of Medicine at Mount Sinai, New York, NY USA; 15 Department of Cell, Developmental, and Regenerative Biology, Black Family Stem Cell Institute, Icahn School of Medicine at Mount Sinai, New York, NY 10029 USA; 16 School of Medicine, Tsinghua University, Beijing, China; 17 AIDS Institute and Department of Microbiology, State Key Laboratory of Emergent Infectious Disease, The University of Hong Kong, Hong Kong, China; 18 Department of Pharmacology and Toxicology, School of Pharmacy, University of Arizona, Tucson, AZ USA

**Keywords:** Viral infection, Embryonic stem cells, SARS-CoV-2, Monocytes and macrophages, Infection

## Abstract

Dysfunctional immune responses contribute critically to the progression of Coronavirus Disease-2019 (COVID-19), with macrophages as one of the main cell types involved. It is urgent to understand the interactions among permissive cells, macrophages, and the SARS-CoV-2 virus, thereby offering important insights into effective therapeutic strategies. Here, we establish a lung and macrophage co-culture system derived from human pluripotent stem cells (hPSCs), modeling the host-pathogen interaction in SARS-CoV-2 infection. We find that both classically polarized macrophages (M1) and alternatively polarized macrophages (M2) have inhibitory effects on SARS-CoV-2 infection. However, M1 and non-activated (M0) macrophages, but not M2 macrophages, significantly up-regulate inflammatory factors upon viral infection. Moreover, M1 macrophages suppress the growth and enhance apoptosis of lung cells. Inhibition of viral entry using an ACE2 blocking antibody substantially enhances the activity of M2 macrophages. Our studies indicate differential immune response patterns in distinct macrophage phenotypes, which could lead to a range of COVID-19 disease severity.

## Abstract (web summary)

Model systems to study SARS-CoV-2 infection are required to better understand the immune response. Here the authors use a lung and macrophage co-culture system by differentiation of human pluripotent stem cells to better understand the phenotype and gene expression changes in host lung cells and macrophages after SARS-CoV-2 infection in vitro.

## Introduction

The infection of severe acute respiratory syndrome coronavirus 2 (SARS-CoV-2) has already caused more than 500 million Coronavirus Disease-2019 (COVID-19) cases internationally (https://google.org/crisisresponse/covid19-map). Most COVID-19 patients show mild to moderate symptoms of fever, dry cough, fatigue, and diarrhea. However, ~15% of confirmed cases progress to severe pneumonia, acute respiratory distress syndrome (ARDS), or multi-organ failure^1^. The progression from mild to severe disease or death is principally attributed to dysfunctional immune responses^2,3^ coupled with viral damage of target cells. However, the reason why only a portion of SARS-CoV-2-infected patients show severe inflammatory conditions has not been clarified yet.

Macrophages are key sentinel cells for host defense in the respiratory system, producing cytokines and chemokines that are crucial components of innate immunity and mediators of immunopathology^4^. Recent studies^5,6^ on the immunity of COVID-19 patients indicated that the cells damaged by SARS-CoV-2 infection triggered innate inflammation in the lungs, which is largely mediated by pro-inflammatory macrophages and granulocytes. In addition to local damage, the pro-inflammatory macrophages release cytokines/chemoattractants and prime adaptive immune cell responses In some cases, this lead to cytokine release syndromes including macrophage activation syndrome (MAS)^7^, followed by respiratory and even multi-organ failure^5^. The systemic cytokine profiles observed in patients with severe COVID-19 were similar to those observed in MAS^2^. These studies imply that macrophages have a crucial function in the progression of SARS-CoV-2 infection.

The activation of macrophages confers a heterogeneous function and plasticity depending on the microenvironment and duration of stimulation, which include a spectrum of phenotypes associated with different inflammatory responses. To facilitate experimental designs, we polarized the macrophages into the M1φ/classically activated and M2φ/alternatively activated^8,9^, even though the distinction is known to be over-simplified since the dynamic activities of macrophages spread along the M1-M2 phenotypic spectrum^10,11^. In general, M1φ cells destroy pathogens by producing a large amount of pro-inflammatory cytokines. In contrast, M2φ cells exhibit anti-inflammatory properties and higher phagocytosis activity against pathogens^12,13^. Therefore, we conducted a series of experiments to investigate the immune response of M1φ, M2φ, or non-activated macrophages (M0φ) to SARS-CoV-2 infection, which could reflect the differential responses seen in patients at different stages of macrophage activation. Despite the approval of some vaccines for disease prevention and emergency use, a deeper understanding of the interactions among targeted cells and SARS-CoV-2 is critically important for studying viral pathobiology and offering better ideas to help combat this deadly contagious disease.

The current most widely used models for SARS-CoV-2 research are the African green monkey derived Vero cells, but these cells still have many limitations for modeling a human disease. Although primary macrophages are more functionally, or phenotypically, representative of the native macrophages of the tissue from which they are derived, they proliferate slowly, are difficult to obtain, and are often poorly characterized^14^. In this study, we generate lung cells and macrophages from the same human pluripotent stem cell (hPSC) lines and pair them to establish a co-culture system. This strategy addresses a common concern about histocompatibility when studying human immune cells with other cell types as well as providing theoretically unlimited cell resources for reliably modeling and studying the immunology of macrophages and human lungs during SARS-CoV-2 infection. Our results demonstrate different immune reaction patterns among distinct phenotypes of macrophages; SARS-CoV-2 infection tends to trigger a cascade of inflammatory pathways and augment inflammation in M1 and M0 macrophages, but not in M2 macrophages. This study also proposes a potential therapeutic strategy through a combination of boosting anti-inflammatory macrophages and blockade of viral entry to control SARS-CoV-2 infection at the immune defense-based protective phase while circumventing the inflammation-driven damaging phase.

## Results

### Involvement of macrophages at the severe stage of COVID-19

**Fig. 1:**
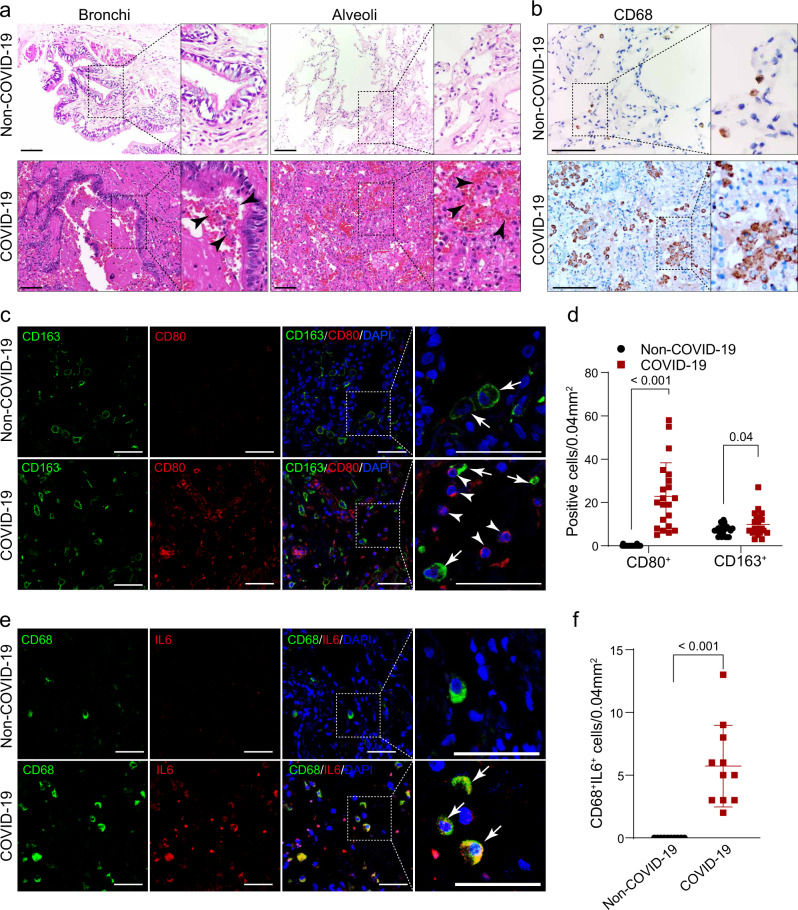
Macrophages were highly involved at the severe stage of COVID-19. **a** H&E (Hematoxylin and Eosin) staining of bronchial or alveolar region in Non-COVID-19 individuals and patients with severe COVID-19. Pulmonary hemorrhagic infarct regions were denoted by arrowheads. Images are representative of three independent experiments. **b** Immunohistochemistry (IHC) using the antibody against CD68 showed macrophages exhibit aggregated phenotype and enlarged nuclei in the lungs of COVID-19 patients, compared to the Non-COVID-19 samples. Images are representative of three independent experiments. **c** Immunofluorescence (IF) staining on Non-COVID-19 or COVID-19 distal lung tissues using antibodies against CD80 (M1 marker) or CD163 (M2 marker). CD163^+^ or CD80^+^ cells were denoted by arrows and arrowheads, respectively. **d** Quantification of CD80^+^ and CD163^+^ macrophages in Non-COVID-19 or COVID-19 distal lung tissues. Data are presented as mean values ± SD. Two-tailed unpaired Student’s t-test. **e** IF staining of non-COVID-19 or COVID-19 distal lung tissues using antibodies against CD68 and IL-6. **f** Quantification of CD68 + and IL-6+ macrophages in Non-COVID-19 or COVID-19 distal lung tissues. Data are presented as mean values ± SD. Two-tailed unpaired Student’s t-test. Scale bars indicate 50 μm in all images in Fig. 1. *n*  =  3 independent experiments.

To better understand how macrophages impact COVID-19 progression, we compared immune cells and inflammatory factors in lung tissues obtained from autopsies of COVID-19 patients and biopsies of non-COVID-19 donors. First, histological changes in lung tissues from COVID-19 patients were examined. Compared to non COVID-19, COVID-19 patients’ bronchioles and alveoli exhibited significant mural edema and lumen occlusion (Fig. 1a). Of note, pulmonary hemorrhagic infarct with abundant inflammatory infiltration (arrowheads) was extensively present throughout the whole alveoli and bronchial regions (Fig. 1a). Recently, it was reported that in the bronchoalveolar lavage fluid, proinflammatory FCN^+^ monocyte-derived macrophages were significantly increased and FABP4^+^ alveolar macrophages (AMs) were greatly reduced in the patients with severe COVID-19, whereas mild and moderate cases were characterized by the presence of highly clonally expanded CD8^+^ T cells^6^. Thus, we examined if macrophages were dominantly presented in the diseased patients’ lungs. Immunostaining against CD68, a pan macrophage marker, showed that macrophages were abundant and extensively distributed throughout the whole lung tissue with aggregated phenotypes (Fig. 1b), in agreement with the above-cited report. However, macrophages are multifaceted, and the distinct functions of macrophages depend highly on their polarization, characterized generally as M1/pro-inflammatory or M2/anti-inflammatory macrophages. We thus further examined M1 macrophage marker CD80 and M2 macrophage marker, CD163. The results show that cells positive for either CD80 or CD163 were aberrantly represented in the patients’ lung tissues (Fig. 1c, d and Supplementary Fig. 1a–d). Indeed, CD68^+^, CD80^+^, and CD163^+^ macrophage populations were significantly expanded in the patients’ lung tissue, suggesting the expansion of both M1 and M2 macrophage populations in severe disease stages. Moreover, we found the CD68^+^FABP4^+^ AMs were significantly decreased in COVID-19 patients’ lungs (Supplementary Fig. 1e, f), which was consistent with the previous report^15^. We also examined several cytokines that are mainly produced by macrophages and found that a cohort of pro-inflammatory cytokines, IL-6, IL-32, CCL2, and IL-1B, were intensively expressed in the lumen of the patients’ lung tissue or in CD68 + macrophages (Fig. 1e and Supplementary Fig. 2a–f). However, we did not find significant differences in CD206^+^HLA-DR^-^ and CD206^-^HLA-DR^+^ macrophages (which represented alveolar and interstitial macrophages, respectively) between COVID-19 and Non-COVID-19 lungs (Supplementary Fig. 1g–i), which represented alveolar and interstitial macrophages, respectively. Taken together, the data suggest the necessity to further examine the functions of macrophages at different activation stages in COVID-19 progression.

### Co-culture of lung cells and macrophages derived from hPSCs

To further investigate the interaction among macrophages, lung cells, and SARS-CoV-2, we established a co-culture model using cells derived from the same hPSC line (RUES2 or H1), which provided a genetically defined background for immune studies. Several effective methods have recently been described^16–18^ for generating major cell types found in human lung tissues by directed differentiation of hPSCs using growth factors and chemicals to alter cell fate-determining signaling pathways. Based on a protocol modified from the ones previously developed by our lab and others^19–21^, hPSCs were differentiated in a stepwise approach into definitive endoderm, anterior foregut endoderm (AFE), lung progenitor cells (LPs), and finally bronchial and alveolar lineage cells (Supplementary Figs. 3a–d, 4a, b). Multiple expected populations that comprised 6 main clusters were identified in the differentiated cells using single cell transcriptomic profiling, including alveolar type II (AT2) cells (*SFTPB*^*+*^*, SFTPD*^*+*^*, SFTPC*^*+*^*, ABCA3*^*+*^), alveolar type I (AT1) cells (*AGER*^*+*^*AQP5*^*+*^), ciliated cells (*FOXJ1*^*+*^*CAPS*^*+*^), stromal cells (*DCN*^*+*^), club cells (*SCGB3A1*^*+*^*SCGB3A2*^*+*^), as well as low numbers of goblet cells (*MUC5AC*^*+*^*MUC5B*^*+*^), basal progenitor cells (*P63*^*+*^), and neuroendocrine cells (*ASCL1*^*+*^*CALCA*^*+*^) (Supplementary Fig. 5a–d). Moreover, the single cell RNA (scRNA) profiles indicated that the hPSC-derived AT2-like and AT1-like cells were enriched with adult human lung AT2 and AT1 cell markers, respectively^22^ (Supplementary Fig. 5g). Correlation analysis of cell fate signature genes further validated the major cell populations including AT1, AT2 cells in the hPSC-lung cells, which showed high similarity to their counterparts in adult human lung^22^ (Supplementary Fig. 5h). ACE2, the putative SARS-CoV-2 receptor, and TMPRSS2, the co-effector for viral entry^23^, were detected in AT2, AT1, and ciliated cells, in clusters 0, 2, and 3, respectively (Supplementary Fig. 5e, f). The immunostaining results further validated that ACE2 was mainly co-expressed with SP-B or pro-SP-C in AT2 cells, and FOXJ1 in ciliated cells (Supplementary Fig. 4b), which is consistent with results previously reported in primary human lung tissues^24^. FACS using anti-ACE2 antibody detected around 20% iLung cells were positive with ACE2 expression (Supplementary Fig. 11a).

**Fig. 2:**
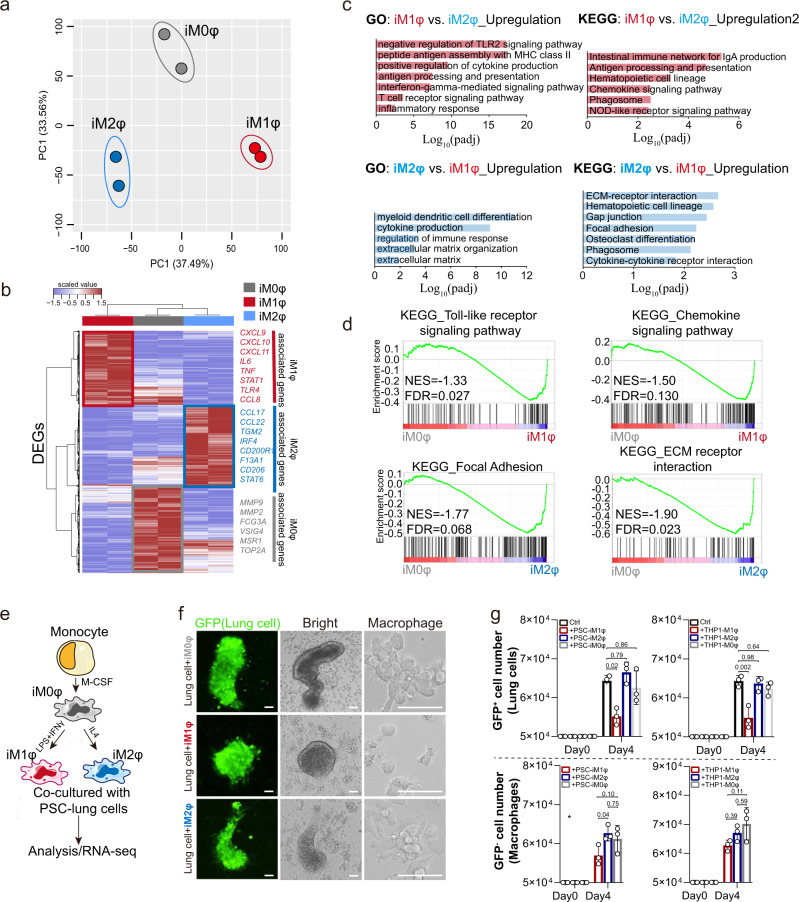
Activation of hPSC-derived macrophages and their co-culture with iLungs. **a**–**d** Bulk RNA sequencing and analysis of the hPSC-derived macrophages, iM0φs, iM1φs, and iM2φs, respectively. **a** PCA plot showing the gene expression profiles of iM0φs, iM1φs, iM2φs. **b** Heatmap of differentially expressed genes (DEGs) in iM0φs, iM1φs, iM2φs. The highly expressed signature genes for each phenotype were highlighted in gray (iM0φ), red (iM1φ) and blue (iM2φ), respectively. **c** GO and KEGG analysis of the genes or pathways that were upregulated or enriched in iM1φs compared with iM2φs and iM2φs compared with iM1φs. **d** Gene Set Enrichment Analysis (GSEA) of KEGG pathways in iM1φs and iM2φs (*P* < 0.05, FDR < 0.25). **e** Schematic of the experimental flowchart of the co-culture systems. **f** Representative bright-field and fluorescence images of the co-culture of lung cells and macrophages derived from hPSC line RUES2. Lung cells are GFP positive. Scale bar = 50 µm. **g** Quantification of lung cells and macrophages (iMφs or THP-1 cells) in the co-cultures of lung cells and iM0φs, iM1φs, iM2φs, and 293T cells. *n*  =  3 independent experiments. Data are presented as mean values ± SD*. p* values were calculated by one-way ANOVA with Tukey’s multiple comparison test.

To generate macrophages and monocytes from hPSCs, we used protocols published by us and others^25–29^. In brief, the hPSCs were first induced to mesoderm and then to vascular mesoderm cells, which were further differentiated into hematopoietic stem and progenitor cells. This was followed with differentiation into functional macrophages by treatment with monocyte inducing cytokines, IL-3, and Macrophage-colony stimulating factor (M-CSF) (Supplementary Fig. 6a–c). The hPSC-induced macrophages (iMφ) expressed major macrophage/monocyte markers, such as CD14, CD11b, and CD68 (Supplementary Fig. 6d, e) and were readily polarized to CD68^+^CD206^+^ macrophages, or CD68^+^FCN1^+^STAT1^hi^ macrophages (Supplementary Fig. 7c) upon stimulation by IL-4 or IFNγ and LPS, respectively (Supplementary Figs. 6h, i, 7a). The iMφs exhibited strong phagocytosis ability when incubated with the GFP-latex beads, which were functionally similar to the primary macrophages (Supplementary Fig. 6f, g). Bulk RNA sequencing (RNA-seq) and analysis were performed to examine the distinct activation of iMφs: classically activated (iM1φ), alternatively activated (iM2φ), or non-activated (naïve, iM0φ). The heatmap and Principal Component Analysis (PCA) plots and heatmap showed differential expression profiles of signature genes associated with M1 or M2 polarization (Fig. 2a, b), such as *IL-6*, *STAT1*, *TLR4*, *CXCL9*, *CXCL10*, and *CXCL11* in iM1φs, and *STAT6*, *IRF4*, *CD206*, *CCL17*, and *CCL22* in iM2φs. Gene Ontology (GO) or Kyoto Encyclopedia of Genes and Genomes (KEGG) enrichment analysis comparing iM1φs, iM2φs, and iM0φs validated the activation of classical signaling pathways such as JAK-STAT and Toll-like receptor signaling pathways in iM1φs; and cell-cell adhesion, osteoclast differentiation, and phagosome related pathways in iM2φs (Supplementary Fig. 8a, b). The comparison between iM1φs and iM2φs indicated that antigen processing and presentation, chemokine signaling pathway, and positive regulation of cytokine production were significantly activated in iM1φs, whereas osteoclast differentiation, myeloid dendritic cell differentiation, and focal adhesion were remarkedly activated in iM2φs (Fig. 2c). Moreover, Gene Set Enrichment Analysis (GSEA) of KEGG signaling pathways underlined that Toll-like receptor and chemokine signaling pathways were highly enriched in iM1φs, indicating a pro-inflammatory state, while adhesion and extracellular matrix receptor-related genes were upregulated in iM2φs, indicating a state of alternative activation (Fig. 2d). Consistent with the previous studies^30,31^, these results suggested that the iMφs derived from hPSCs were physiologically functional and capable of polarizing into distinct pro- or anti-inflammatory phenotypes, inducing inflammatory responses, and upregulating expression of a variety of cytokines and chemokines upon stimulation.

Next, the hPSC-derived lung cells (iLungs) and macrophages (iMφs) were plated and cultured together in a 1:1 ratio (Fig. 2e), similar to the ratio of lung cells and macrophages in distal bronchial or alveolar regions in the human lungs^22^. The iLungs was derived from the hPSC lines carrying a doxycycline-inducible GFP reporter gene, which allowed the distinction of iLungs and iMφs in live cultures (Fig. 2f). A significantly lower number of GFP^+^ iLungs were observed after four days of co-culture with iMφs of M1 phenotype (iM1φs) compared to the co-culture with iMφs of M2 phenotype (iM2φs), M0 phenotype (iM0φs) or control 293T cells (Fig. 2g).

The scRNA profiling was applied to further characterize the co-cultures (Supplementary Figs. 7, 9, 10a–c). The analysis showed decreased expression of proliferation-associated genes *MKI67 and TOP2A* and increased expression of apoptosis-related genes *TP53*, *CASP3*, *BAX*, *MCL1*, in the iLungs co-cultured with iM1φs, but not in that co-cultured with iM2φs (Supplementary Figs. 7b, 10f). These results aligned with the phenotype of pro-inflammatory activities of iM1φs, as both bulk RNA-seq and scRNA-seq data detected a set of pro-inflammatory factors, *IL-1B*, *IL-18*, *STAT1*, *FCN1*, *CXCL9*, *CXCL10*, *CXCL11*, *CXCL16*, and *CCL2* highly expressed in iM1φs (Supplementary Figs. 7b, c, 9a, b). In contrast, iM2φs mainly expressed anti-inflammatory factors or immunoregulatory genes such as *TGM2*, *APOE*, *A2M*, *CCL13*, *CCL26*, and *TREM2* (Supplementary Figs. 7b, c, 9a, b). GO enrichment analysis comparing iM1φs and iM2φs showed over-activation of differential signaling pathways such as the cellular response to IFNγ and the defense response to viruses in iM1φs, while anti-inflammatory, tissue damage-repair process of RNA catabolic process and protein co-localization to endoplasmic reticulum pathways were upregulated in iM2φs (Supplementary Fig. 10d, e). Similar phenotypes were observed in the iLungs co-cultured with THP-1 cells, an established monocyte line, upon activation of the M1 or M2 phenotype (Fig. 2g). Our results are consistent with previous studies showing that M1 macrophages may decrease the viability of lung cells by producing inflammatory cytokines^32,33^. These results indicated that activation of M1-macrophage was sufficient to create a toxic environment for the iLungs, even in the absence of viral infection.

### Immune response of macrophages following SARS-CoV-2 infection

**Fig. 3:**
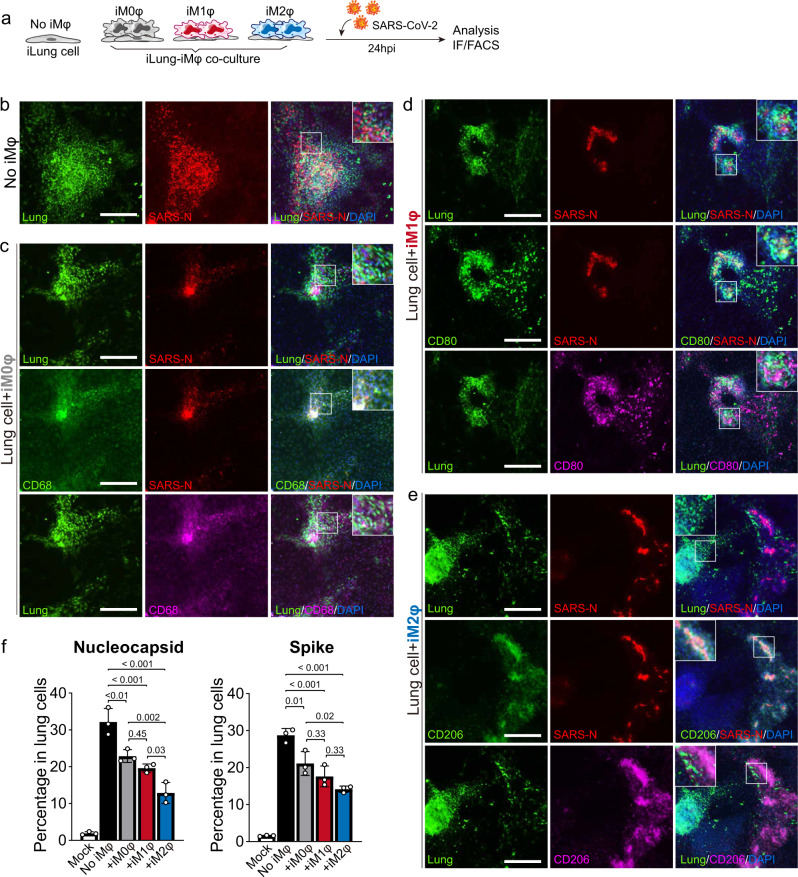
Differential responses of the iM0φ, iM1φ and iM2φ to SARS-CoV-2 infection. **a** Schematic of the experimental flowchart of SARS-CoV-2 infection of the co-culture systems. **b**–**e** IF staining of the co-cultures of iLungs with 293 T cells (**b**), iM0φs (**c**), iM1φs (**d**), and iM2φs (**e**) infected with SARS-CoV-2 at 24 hpi (MOI = 0.1) and mock samples, using antibodies detecting SARS-CoV-2 nucleocapsid protein (SARS-CoV-2), CD68 (M0φ), CD80 (M1φ), and CD206 (M2φ). iLung cells expressed GFP. Scale bar = 100 µm. **f** Quantification of infected lung cells in the co-cultures at 24 hpi by FACS using antibodies against SARS-2 N and S protein. *n*  =  3 independent experiments. Data are presented as mean values ± SD. *p* values were calculated by one-way ANOVA with Tukey’s multiple comparison test.

To model the immune response of macrophages to SARS-CoV-2 infection in lung cells, the virus was introduced to the co-culture system (Fig. 3a). After 24 h of incubation with the SARS-CoV-2 virus (USA-WA1/2020, MOI = 0.1), a significantly greater decrease in the amount of viral proteins (SARS-CoV-2 nucleocapsid (N) proteins) was observed in the co-culture of iLungs and iMφs compared to the co-culture of iLungs and 293T cells (293T cells were used as a co-culture control, based on our preliminary data and previous report that the permissiveness of 293T to SARS virus is low^32^). Strikingly, most viral proteins were detected in the iM2φs when co-cultured with iLungs, while substantial levels of viral proteins were detected in iLung cells in the co-cultures with iM1φs, iM0φs, and 293T cells (Fig. 3b–e). These results were further validated by flow-cytometry analysis using an antibody against SARS-2 N or spike (S) protein, showing that the iL–ungs co-cultured with iM2φs had lower infection rates of iLung cells by SARS-CoV-2, compared to the co-cultures with iM1φs, iM0φs, and 293 T cells (Fig. 3f). In agreement with this phenotype, the RNA-seq analysis detected higher viral loads in the iM2φs than in iM1φs or iM0φs, after incubating iMφs with SARS-CoV-2 for 24 h (Supplementary Fig. 12a, b). In addition, the expression of ACE2 and TMPRSS2 was undetectable in the bulk RNA-seq of iMφs (Supplementary Fig. 12c), and very few iMφs expressed ACE2 and TMPRSS2 based on scRNA profiling (Supplementary Fig. 9b). The lack of ACE2 expression in iMφs was also validated with FACS (Supplementary Fig. 11a–c), indicating iMφs were not permissive to SARS-CoV-2, and the viral proteins detected in iMφs were mainly due to uptake of virus or other infected cells.

**Fig. 4:**
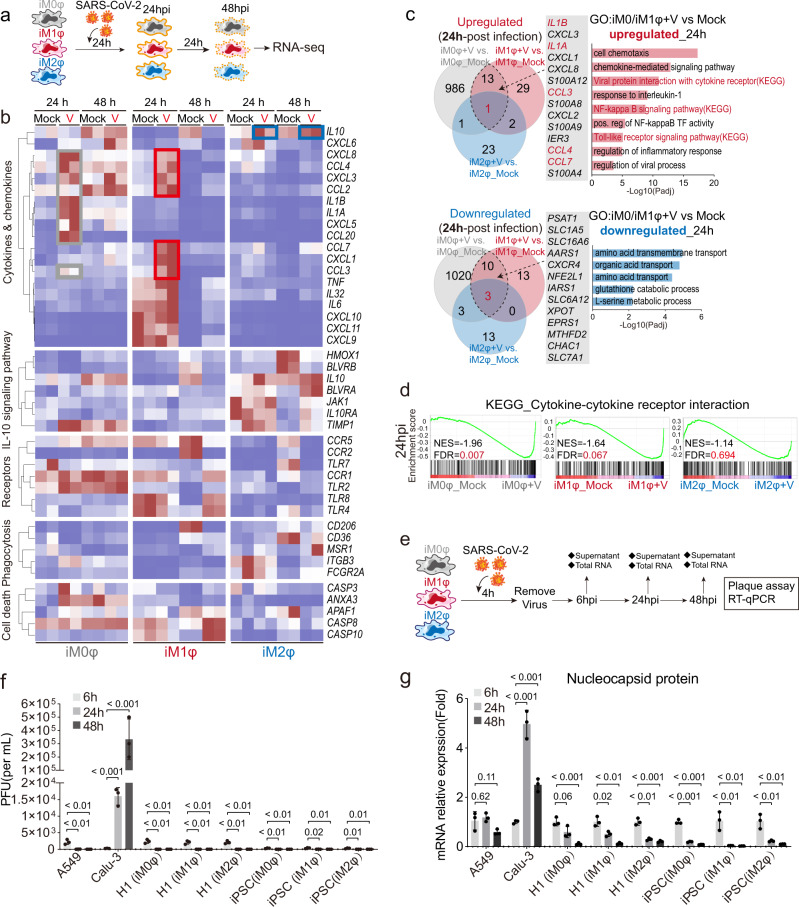
Immune reaction of macrophages following SARS-CoV-2 infection. **a** Schematic of the experimental flowchart of SARS-CoV-2 infection of macrophages. **b** Heatmap analysis of cytokines, chemokines, receptors, phagocytosis and cell death related genes in iM0φ, iM1φs and iM2φ cells at 24hpi or 48 hpi. **c** Venn plot comparing the overlap of upregulated and downregulated genes upon SARS-CoV-2 infection in iM1φ and iM0φ cells at 24hpi. Bar graph showing the GO enrichments of the overlap genes. **d** GSEA diagrams comparing the cytokine–cytokine receptor interaction following SARS-CoV-2 infection in iM0φ, iM1φ, and iM2φ at 24hpi. **e** Schematic of the experimental design of the macrophages on SARS-CoV-2 infection for plaque assay and RT-qPCR. **f** Comparative graph of plaque assay results in A549(negative control), Calu-3(positive control), H1-ESC-derived iMACs (H1-iM0φs/iM1φs/iM2φs) and iPSC-derived iMACs (iPSC-iM0φs/ iM1φs/iM2φs) on SARS-CoV-2 infection at 6,24 and 48 hpi, respectively. Virus was only detected in the supernatant of Calu-3 cells. *n*  =  3 independent experiments. Data are presented as mean values ± SD. Statistically significant differences are calculated using an unpaired two-tailed unpaired Student’s *t* test. **g** Bar graph depicts RT-qPCR analysis of mRNA expression of SARS-CoV-2 nucleocapsid in control cells and iMACs on SARS-CoV-2 infection at 6,24 and 48 hpi, respectively. Increased mRNA of virus was only detected in Calu-3 cells. *n*  =  3 independent experiments. Data are presented as mean values ± SD. Statistically significant differences are calculated using an unpaired two-tailed unpaired Student’s t-test.

Next, bulk RNA-seq was performed to thoroughly examine the immune response in iMφs after SARS-CoV-2 infection (Fig. 4a). Heatmap analysis indicated that a cohort of cytokines, chemokines, and inflammatory factors were substantially upregulated in iM1φs and iM0φs 24 h after incubation with the SARS-CoV-2 virus (Fig. 4b and Supplementary Fig. 14d). The majority of upregulated cytokines and chemokines, such as *IL-6, TNF, CCL2, CCL3*, and *CXCL10*, have been reported to be associated with cytokine release syndrome (CRS), including MAS, in severe COVID-19 cases. Gene enrichment analyses comparing iMφs incubated with virus and the mock indicated over-activation of differential signaling pathways that are known to be important for innate or adaptive immune responses, such as chemokine-mediated signaling pathway, TOLL-like receptor signaling, and response to IL-1 in iM1φ (Fig. 4c and Supplementary Fig. 14b); cellular chemotaxis, JAK-STAT, and NF- κB signaling pathways in iM0φ (Fig. 4c and Supplementary Fig. 14b). GSEA analysis found that JAK-STAT and TOLL-like receptor signaling pathways remained activated in iM0φs, iM1φs and iM2φs at 48 hpi (Supplementary Fig. 14a). The viral infection also led to common alterations in some genes and signaling pathways in both iM1φ and iM0φ cells, such as *IL1A*, *IL1B*, *CCL7*, chemokine-mediated signaling, viral protein interaction with cytokine–cytokine receptor, and Toll-like receptor signaling (Fig. 4c). These results suggested that both iM1φs and iM0φs could significantly induce or enhance inflammatory reaction following SARS-CoV-2 infection. In addition, the previous studies by us^33^ and others^34^ suggested that lung cells display self-immune defense after SARS-CoV-2 infection, releasing inflammatory factors, such as CXCL2, CCL2, CXCL3, and IL-1A, as well as BCRC3, AADAC, and ATPB4, which combined with the pro-inflammatory reaction of M1 or M0 macrophages. This process could lead to further pulmonary inflammation and damage (Fig. 4b).

Unlike iM1φs or iM0φs, iM2φs demonstrated mild inflammatory reaction upon SARS-CoV-2 infection. As no substantial change was observed in the expression of main inflammatory factors (Fig. 4b), differentially activated signaling pathways were focused on response to virus and phagocytosis, and genes related to pro-inflammatory activation were downregulated (Supplementary Fig. 14c). Moreover, IL-10 signaling-related genes, such as *IL10, IL10RA, HMOX1, JAK1*^35^, and *BLVRA/B*, were enriched in iM2φs, suggestive of anti-inflammatory macrophages (Fig. 4b). Besides, phagocytosis or anti-viral activity-related genes (such as *MSR1, CD36*) or viral protein interactions with cytokine-cytokine receptors, were upregulated in iM2φ following viral infection (Fig. 4b). GSEA analysis of the KEGG signaling pathway indicated that genes related to the cytokine-cytokine receptor interaction were highly enriched in iM0φs and iM1φs at 24 h post infection, whereas they were not enriched in iM2φs, indicating weaker immune responses in iM2φs, compared with iM0φs and iM1φs (Fig. 4d). In summary, these results demonstrated a differential immune response of iM2φs versus iM1φs or iM0φs upon SARS-CoV-2 infection. iM2φs increased phagocytosis activity and upregulated anti-inflammatory factors, while iM1φs and iM0φs increased antigen-presenting activity and upregulated pro-inflammatory factors.

Furthermore, a cohort of cell death-related genes, such as *CASP3*, *CASP 8*, *CASP 10*, and *APAF1*, were markedly upregulated in iM1φs or iM0φs incubated with SARS-CoV-2 (Fig. 4b). These results indicated that cell death or decrease in cell viability may occur in iM1φs or iM0φs, especially 48 h after infection; this phenotype could attribute to viral damage or hyper-inflammatory reaction of macrophages upon infection. In contrast, no significant alteration of the aforementioned cell death-related genes or signaling pathways was observed in iM2φs following viral infection (Fig. 4b). Altogether, these findings suggest that activation of pro-inflammatory macrophages can aggravate damage in lung cells as well as macrophages beyond the destruction by viral infection, while activation of anti-inflammatory macrophages provides a protective effect for lung cells and macrophages from viral infection.

### hPSC-derived macrophages are not permissive to SARS-CoV-2 infection

To investigate the dynamics of viral RNA and protein in iMφs after infection, we compared the cell type specific permission upon viral infection. Polarized or non-polarized iMφs were incubated with SARS-CoV-2 for 4 h followed by the collection of the supernatant and extraction of the RNA from infected cells at 6, 24, and 48h-post infection for downstream analysis (Fig. 4e). We found the virus was only detectable in the supernatant of Calu3 cells, but not found in any type of iMφs derived from hESCs or hiPSCs, indicating the virus could not release from the iMφs (Fig. 4f). The viral RNA was uniformly decreased in iMφs but was only increased in Calu3 cells in the first 24 h and a large amount of viral RNA were detectable at 48 h-post infection (Fig. 4g, and Supplementary Fig. 13a). We next examined the location of a viral protein inside the cells by co-staining with a lysosome marker, LAMP-2. We detected SARS-N protein in all types of cells except A549 cells, indicating the viruses could enter Calu3 cells and iMφs, and had different ratios of overlapped regions (Supplementary Fig. 13b). Taken together, these results suggested that the uptake of SARS-CoV-2 by iMφs is ACE2- independent.

### Blockage of ACE2 enhances elimination of SARS-CoV-2 by macrophages

**Fig. 5:**
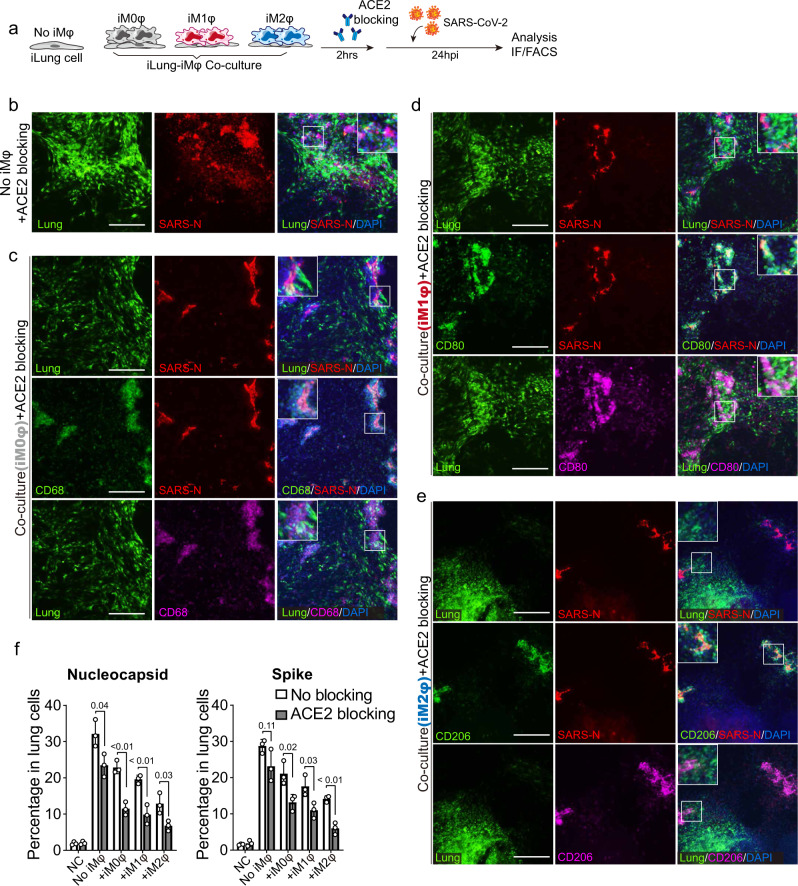
Blockage of ACE2 enhanced the elimination of SARS-CoV-2 by macrophages. **a** Schematic of the experimental flowchart showing the infection of co-cultures with SARS-CoV-2. The Immunofluorescence analysis of the SARS-CoV-2 infected co-culture systems in the presence or absence of ACE2 blocking antibody. ACE2 blockage antibody was applied two hours prior to the virus addition and IF staining was performed on the co-cultures of lung cells and 293 T (**b**), iM0φs (**c**), iM1φs (**d**), and iM2φs (**e**) that were infected with SARS-CoV-2 and mock samples at 24 hpi (MOI = 0.1), using antibodies against SARS-CoV-2 nucleocapsid protein (SARS-CoV-2), CD68 (M0φ), CD80 (M1φ), and CD206 (M2φ). Lung cells expressed GFP. Scale bar = 100 µm. **f** Comparison of the percentages of infected lung cells with or without ACE2 blocking at 24 hpi using FACS. *n*  =  3 independent experiments. Data are presented as mean values ± SD. *p* values were calculated by two-tailed unpaired Student’s *t* test.

Several studies^36^ on mild or recovered COVID-19 cases indicated that neutralizing antibodies produced in individuals with a healthy immune response can block viral infection. Antibody production is followed by alveolar macrophages recognizing the neutralized viruses and clearing them by phagocytosis. We sought to model this process using an ACE2 blocking antibody to inhibit virus entry to target cells, thereby decreasing the viral load (Fig. 5a), to test whether this enhances phagocytic activity of macrophages. As expected, incubation with ACE2 blocking antibody two hours before infection with SARS-CoV-2 virus markedly reduced the viral protein (SARS-CoV-2 N protein) in the co-cultures of iLungs with iMφs or 293T cells, compared to those co-cultures without ACE2 blocking (Fig. 5f). Immunostaining results showed that iM2φs had the most SARS-CoV-2- N proteins, not in the iLungs, while the N protein was clearly found in iLungs co-cultured with iM1φs, iM0φs, and 293T cells (Fig. 5b–e). Similarly, flow-cytometry analysis validated that the decrease of lung infection was most pronounced in the co-cultures with iM2φs, compared to those co-cultured with iM1φs, iM0φs, or 293T (Fig. 5f).

These results demonstrated that early intervention of viral infection by blocking ACE2 in target cells can increase the clearance of virus by macrophages. Synergizing this approach with the phagocytosis activity of M2-macrophages provided further protection for target cells and reduce the damage by inflammatory factors produced by M1-macrophages.

## Discussion

The study of human host-immune systems with pathogens has been historically dependent on the use of animal models, largely due to limited human tissue derived cell resources. Immune research on COVID-19 is limited by the types of models available for study. Recently, a transgenic mouse strain^37^ has been made with human ACE2 expression regulated by a human cytokeratin-18 promoter, but the ACE2 expression in humans is more complex than its expression in the mice. Another model is the ferret^38^, which can be infected with SARS-CoV-2, but does not develop hyper-inflammation in the lung. Recent advances in stem cell biology, especially the technology of human pluripotent stem cells (hPSCs) differentiation into functional immune cell types, provides a rigorous human model system for studying immunology and therapeutics. In this report, we describe a co-culture system in which the immune cells, specifically monocytes/macrophages and lung lineage cells are produced by directed differentiation of hPSCs. Several key features make the human cell model an ideal system for studying the immunology of SARS-CoV-2. The model contains the host cells and immune cells from the same hPSC lines, avoiding concerns of histocompatibility, while it can provide abundant numbers of cells with a genetically defined background for robust mechanistic or therapeutic studies.

The innate immune response mainly mediated by macrophages or granulocytes, responding to tissue damage caused by SARS-CoV-2 infection, likely contributes to the ARDS, which is characterized by the rapid onset of widespread inflammation in the lung and subsequent respiratory failure^5^. Our study of COVID-19 patients’ lung biopsies validated a correlation between the macrophages and the disease, showing a heavy infiltration of pro-inflammatory macrophages in tissue samples from distal lung regions with high levels of inflammatory cytokine IL-6 in severe cases. The macrophage and lung cell co-culture model combined with transcriptomics and other analyses were then applied to interrogate the immune responses of distinct phenotypes of macrophages following SARS-CoV-2 infection. We discovered that pro- and anti-inflammatory macrophages both have similar capacities to eliminate the virus in the context of a moderate viral load. However, the immune reaction of pro-inflammatory macrophages led to more damage on lung cells and secretion of a set of inflammatory factors, including IL-6, TNF, CCL2, CCL3, and CXCL10 that are known to be mediators in dysfunctional immune responses and CRS. Similarly, the naïve macrophages became activated in response to the viral infection and expressed essential inflammatory molecules like IL-1β, CCL3, and CCL4. In contrast, anti-inflammatory macrophages protected lung cells from viral infection and diminished pulmonary inflammation by phagocytosis and production of anti-inflammatory factors related to IL-10 signaling pathway. Finally, inhibiting viral entry into target cells using an ACE2 blocking antibody diminished viral infection and enhanced the elimination of viruses. In particular, the intervention on viral entry can synergize with the phagocytosis and antiviral activity of macrophages, resulting in a more pronounced clearance of virus and protection of target cells.

Accumulating evidence has indicated that the cytokine storm, particularly MAS, is often associated with COVID-19 pneumonia and its exacerbation in severe cases^36^. Our co-culture model of lung cells with distinct phenotypes of macrophages could recapitulate the main features of immune response in different activation stages of macrophage in COVID-19 patients. We found that SARS-CoV-2 infection induced or strengthened inflammatory reaction in M0 or M1 macrophages, leading to expression of a cohort of inflammatory factors with similarity to those observed in patients with severe COVID-19 and CRS^39^. On the other hand, M2 macrophage exhibited a minimal inflammatory response following SARS-CoV-2 infection, as no significant increase was observed in the expression of inflammatory cytokines and chemokines. These differential immune response patterns of macrophages may partially explain the variation in COVID-19 susceptibility and symptoms. For instance, certain preexisting conditions like hypertension or diabetes, with increased M1/M2 ratio of macrophages, are considered risk factors for severe COVID-19; other conditions such as allergic asthma^40^, favoring M2 activation, are less likely to be associated with severe COVID-19. In addition, our results are in line with the current evidence^6^ that pathological macrophages infiltrating the lungs and other organs in severe cases were mostly derived from peripheral circulating monocytes rather than from tissue-resident macrophage types, which tend to be alternatively activated. Taken together, our results manifest the difference in macrophage activation could be a contributing factor to the variation in COVID-19 symptoms; however, the exact drivers of macrophage activation and molecular events behind their phenotype need further investigation in patients.

## Methods

### Settings

The study was performed on paraffin-embedded lung tissues of COVID-19 patients and non-COVID-19 controls, were acquired from the department of pathology in the 3rd people’s hospital of Shenzhen, China. All research activities with human lung specimens of COVID-19 patients and non-COVID-19 controls were implemented under the protocol approved by the institutional research ethics committee of 3rd hospital of Shenzhen (No. 2021-0089). Informed consent was obtained from the patients and families. Participants were not compensated.

All embryonic stem cell studies were approved by the Institutional Review Board (IRB, IRB20-0598) at the University of Chicago, or by the Tri-Institutional ESCRO committee (Weill Cornell Medicine, Memorial Sloan Kettering Cancer Center, and Rockefeller University).

### Key resources

All the growth factors, antibodies, chemicals, peptides, recombinant proteins, cell lines, and culture medium are provided in Supplementary Table 1.

### Patient’s lung tissues

The paraffin-embedded lung tissues were acquired from the department of pathology in the third people’s hospital of Shenzhen, China. They reported the pathological changes of lungs in critical COVID-19 infection^41^ (a 66 year-old male, a 62 year-old female, and a 31 year-old male; all patients are Asian Chinese). The diagnosis of COVID-19 pneumonia was based on the “Coronavirus Pneumonia Prevention and Control Plan” (7th edition) newly issued by the National Health Commission, China^42^. Nasopharyngeal swabs were collected and COVID-19 was detected by real-time polymerase chain reaction. Infection was defined as at least two positive test results. The COVID patients developed respiratory failure and septic shock during the treatment. Informed consent was obtained from the patients and families.

For non-COVID-19 paraffin-embedded lung tissues, the samples were obtained from two patients undergoing elective surgery who were diagnosed with chronic bronchitis (a 47-year-old male, Asian Chinese) or lung cancer before chemotherapies (a 59-year-old female, Asian Chinese) respectively. Diseased lung tissues were removed as part of routine clinical care but surplus for routine diagnostic requirements. This study was approved by the Institutional Review Board of the third People’s hospital of Shenzhen (2021-0089). Surgical informed consent was obtained from patients or guardians before the sample collection.

### hPSC lung differentiation

Protocols for maintenance of hPSCs and generation of lung cells were slightly modified from previous studies^20,21^. The hESC line-RUES2 and H1 are both purchased from WiCell and were cultured on irradiated mouse embryonic fibroblasts (Global Stem) at a density of 20,000–25,000 cells/cm^2^ in a medium of DMEM/F12, 20% knockout serum replacement (Life Technologies), 0.1 mM β-mercaptoethanol (Sigma Aldrich) and 20 ng/ml bFGF (R&D Systems), and medium was changed daily. hESC cultures were maintained in an undifferentiated state at 37 °C in a 5% CO_2_/air environment until stem cells reached about 90% confluence.

hESC differentiation into endoderm was performed in serum-free differentiation (SFD) medium of IMDM/F12 (3:1) (Life Technologies) supplemented with N2 (Life Technologies), B27 (Life Technologies), 50 μg/ml ascorbic acid, 2 mM Glutamax, 0.4 μM monothioglycerol, and 0.05% BSA at 37 °C in a 5% CO_2_/5% O_2_/95% N_2_ environment. hESCs were treated with Accutase (Stemcell Technologies) and plated onto low attachment six-well plates (Corning Incorporated), resuspended in endoderm induction medium containing 10 μM Y-27632, 0.5 ng/ml human BMP-4, 2.5 ng/ml human bFGF, and 100 ng/ml human Activin A, for 72–76 h dependent on the formation rates of endoderm cells. On day 3, the endoderm bodies were dissociated into single cells using 0.05% Trypsin/0.02% EDTA and plated onto fibronectin-coated, 24-well tissue culture plates (~100,000–150,000 cells/well). For induction of AFE, the endoderm cells were cultured in SFD medium supplemented with 1.5 μM Dorsomorphin dihydrochloride (R&D Systems) and 10 μM SB431542 (R&D Systems) for 48 h, and then switched to 24 h of 10 μM SB431542 and 1 μM IWP2 (R&D Systems) treatment. For induction of early-stage lung progenitor cells (day 6–15), the resulting anterior foregut endoderm was treated with 3 μM CHIR99021, 10 ng/ml human FGF10, 10 ng/ml human FGF-7, 10 ng/ml human BMP-4 and 50-60 nM all-trans retinoic acid (ATRA), in SFD medium for 8–10 d. The day 10-15 cultures were maintained in a 5% CO_2_/air environment. On days 15 and 16, the lung progenitor cells were replaced after 1-min trypsinization onto fibronectin-coated plates, in the presence of SFD containing 3 μM CHIR99021, 10 ng/ml human FGF10, 10 ng/ml human FGF7, in a 5% CO_2_/air environment. For differentiation of mature lung cells (day 25–55), cultures were re-plated after brief trypsinization onto 2D 3.3% Matrigel-coated 24-well plates in SFD media containing maturation components containing 3 μM CHIR99021, 10 ng/ml human FGF-10; 10 ng/ml human FGF-7, and DCI (50 nM Dexamethasone, 0.1 mM 8-bromo-cAMP (Sigma Aldrich) and 0.1 mM IBMX (3,7-dihydro-1-methyl-3-(2-methylpropyl)-1H-purine-2,6-dione) (Sigma Aldrich)). The protocol details are summarized in Supplementary Fig. 2a.

All embryonic stem cell studies were approved by the Institutional Review Board (IRB) at the University of Chicago, or by the Tri-Institutional ESCRO committee (Weill Cornell Medicine, Memorial Sloan Kettering Cancer Center, and Rockefeller University).

### hPSC macrophage differentiation

We derived macrophages from hESC line H1 or human iPSC line IMR90-1 and adapted based on previously reported protocols^25,26,28^. For macrophage differentiation, at day -2, hESCs were digested into single-cell suspension by 1 mg/ml Accutase (Stemcell Technologies) and plated onto Matrigel-coated culture dishes at a density of 2 × 10^4^ cells/cm^2^ in mTeSR1 medium (Stemcell Technologies) with 5 μM Y27632 (MedchemExpress). After 24 h, Y27632 was withdrawn from the medium and cells were cultured for another 24 h. At day 0, cells were firstly induced by macrophage differentiation basal medium (SFD-M) which is RPMI 1640 medium supplemented with 2% B27 (Thermo Fisher Scientific), 1% L-GlutaMAX-I and 50 μg/ml ascorbic acid (Sigma Aldrich) and 10 ng/ml BMP4 (R&D Systems) for 24 h. Afterward, the medium was changed to SFD-M medium containing 10 ng/ml BMP4 and 2 μM GSK3 inhibitor CHIR99021 (Cayman Chemical) for another 48 h. On day 3, cells were replated onto Matrigel-coated dishes at a density of 4 × 10^4^ cells/ cm^2^ in SFD-M medium with 50 ng/ml VEGF (R&D Systems) and 10 ng ng/ml FGF2 (R&D Systems) for 48 h. On day 5, the medium was replaced with basal medium with 50 ng/ml VEGF, 10 ng ng/ml FGF2 and 10uM TGFβ inhibitor SB431542 (R&D Systems) for another 72 h. On day 8–10, floating cells were collected and medium was changed and supplemented with 50 ng/ml M-CSF and 10 ng/ml IL-3 (R&D Systems) for another 3-5 days. From day 11-13 onward, the medium was changed to SFD-M medium with 50 ng/ml M-CSF for 3 days. All differentiation steps were cultured under normoxic conditions at 37 °C, 5% CO_2_. The protocol details are summarized in Supplementary Fig. 4a.

All embryonic stem cell studies were approved by the IRB at the University of Chicago, or by the Tri-Institutional ESCRO committee (Weill Cornell Medicine, Memorial Sloan Kettering Cancer Center, and Rockefeller University).

### hPSC monocyte polarization and co-culture with lung cells

hPSC-derived CD14^+^ cells were plated on tissue culture plates at a density of 2 × 10^4^ cells/cm^2^ in SFD-M medium supplemented with 50 ng/mL M-CSF. After 2 days of culture, monocytes differentiated into M0 macrophages and polarized to M1 or M2 macrophages. For macrophages polarization, 100 ng/mL LPS (Sigma-Aldrich) and 10 ng/mL IFNγ (R&D Systems) were added for M1 macrophage induction, or 20 ng/m IL-4 (R&D Systems) was added for M2 macrophage induction in SFD-M medium supplemented with 50 ng/mL M-CSF, respectively. These cells were cultured for another three days before examination for expression of the M1 or M2 macrophage markers.

For the co-culture of ilung cells with iMφs, the 2D culture of lung cells were dissociated into single cells or small clusters by digesting with trypsin for 7–8 min, and seeded at 5 × 10^4^ cells/ well, on 3.3% Matrigel-coated 12-well plates. After 6 hours incubation in a 37 ^o^C, 5% CO_2_/air environment, iM0φ, polarized iM1φ or iM2φ were seeded at 5 × 10^4^ cells/ well on the same wells with ilung cells, and the cells were cultured for another 4 days in the mixture media of 1:1 ilung media: iMφ media. Because iLung cells are GFP positive, the cell percentages and numbers of ilung and iMφ were calculated by FACS.

### Giemsa staining

Differentiating day 11–13 monocytes/macrophages were fixed on slides using Cytospin, followed by staining using Wright-Giemsa Stain (Sigma-Aldrich) according to the manufacturer’s instructions.

### Phagocytosis assay

Differentiating day 14 macrophages were dissociated by accutase and replated in 24-well plates at a density of 1 × 10^5^ /ml in RPMI 1640 medium supplemented with 1% penicillin–streptomycin, 2 mM GlutaMAX, 10% FBS and 50 ng/ml M-CSF. The next day, GFP-labeled latex beads (1.0 μm mean particle size, Sigma) were added at a ratio of iMACs: beads = 1:10. Two hours later, iMACs were rinsed gently with PBS twice and quantified by flow cytometry.

### Immunohistochemical staining

For immunohistochemical staining, paraffin-embedded sections were deparaffinized and incubated with primary antibodies at 4 °C overnight and secondary antibodies at room temperature for 1 h. Primary antibodies and secondary antibodies are described in the supplementary Table. Nuclei were counterstained by Hoechst 33342 (Sigma-Aldrich). positive cells in lungs were randomly counted from different visions of slides by confocal microscopy. 12 views in each lung section were counted and averaged cell numbers per 0.04 mm^2^ were used to define the distributions of positive cells in the lung tissues^43^. Living cells in culture were directly fixed in 4% paraformaldehyde for 25 min, followed with 15 min permeabilization in 1% triton X-100. For immunofluorescence, cells or tissue sections were immunostained with antibodies and counterstained with 4,6-diamidino-2-phenylindole. Adjacent sections stained with H and E were used for comparison. The antibodies used for immunostaining experiments are listed in Supplementary Table 1.

### Fluorescent activated cell sorting (FACS)

For extracellular staining, basically cells were resuspended in a FACS buffer (PBS with 0.1 % BSA and 2.5 mM EDTA) and incubated with antibodies for 30 min at 4 °C, followed with washed and suspended in 0.1% BSA/PBS buffer. For intracellular staining, cells were fixed with 2% paraformaldehyde for 20 mins at room temperature. For permeabilization, cells were resuspended in 0.2% Triton-X100 in PBS for 10 min, followed with washed and suspended in 0.1% BSA/PBS buffer, the following procedures were the same as the extracellular staining. Negative controls stained with control IgG instead of primary antibodies were always performed with sample measurements. Flow cytometry machine of BD BD LSR Fortessa and software of FlowJo (Version x.0.7) were mainly used to collect and analyze the flow cytometry data.

### SARS-CoV-2 infections

SARS-CoV-2, isolate nCoV/Washington/1/2020 (kindly provided by the National Biocontainment Laboratory, Galveston, TX), was propagated in Vero E6 cells in DMEM supplemented with 2% FBS, 4.5 g/L D-glucose, 4 mM L-glutamine, 10 mM Non-Essential Amino Acids, 1 mM Sodium Pyruvate and 10 mM HEPES^44^. hESC-derived lung and macrophage co-cultures or macrophages alone in 24-well plates were infected with SARS-CoV-2 for 24 h or 48 h at a MOI of 0.1 in the medium containing SFD: SFD-M = 1:1. For RNA-seq, cells were washed three times in PBS and lysed with TRIzol for 30 min, after which samples were tested for viral inactivation before being removed from the BSL-3 facility for RNA extraction and sequencing.

All work involving live SARS-CoV-2 was performed in the CDC/USDA-approved BSL-3 facility at the Howard Taylor Ricketts Laboratory of the University of Chicago at Argonne National Laboratory in accordance with institutional biosafety requirements.

### SARS-CoV-2 infection for plaque assay

The SARS-CoV-2 isolate HK-95 (accession no. MT835143) were isolated from specimens obtained from five laboratory-confirmed COVID-19 patients using Vero E6 cells. All experiments involving SARS-CoV-2 viruses were conducted in a Biosafety Level-3 laboratory. Virus titers were determined by plaque assay using Vero E6 cells. Macrophages, including M0, M1, and M2, were infected by virus with 0.1 of MOI, using A549 and Calu-3 cell lines as negative and positive controls. Virus infected cells were cultured at 37 °C. For plaque assay, the supernatant was harvested at the indicated time points in Vero E6 cells to determine the virus titer in supernatant, and the cells were lysed by TRIzol reagent for RNA extraction after the flushing of PBS. The mRNA expression levels of N- and S-protein were detected by RT-qPCR. The primers for SARS-N: forward: GCCTCTTCTCGTTCCTCATCAC, reverse: AGCAGCATCACCGCCATTG; For SARS-S: forward: CTTCCCTCAGTCAGCACCTC, reverse: AACCAGTGTGTGCCATTTGA. All work involving live SARS-CoV-2 was performed in the BSL-3 facility, State Key Laboratory of Emergent Infectious Disease, at the University of Hong Kong.

### Real-time quantitative PCR

RNA was extracted using Direct-zol RNA Miniprep kit (Zymo Research). RNA concentrations were measured using the NanoDrop system (Thermo Fisher Scientific). cDNA was synthesized using PrimeScript RT Reagent Kit with gDNA Eraser. mRNA expression was determined by QPCR in a CFX96 thermal cycler (Biorad). The primers for SARS-N: forward: GCCTCTTCTCGTTCCTCATCAC, reverse: AGCAGCATCACCGCCATTG; For SARS-S: forward: CTTCCCTCAGTCAGCACCTC, reverse: AACCAGTGTGTGCCATTTGA.

### Bulk RNA sequencing of hPSC-derived macrophages

Total RNA was isolated using Direct-zol RNA Miniprep kit (Zymo Research). Following RNA isolation, total RNA integrity was checked using a 2100 Bioanalyzer (Agilent Technologies). RNA concentrations were measured using the NanoDrop system (Thermo Fisher Scientific). Preparation of RNA sample library and RNA-seq were performed by the Genomics Core Laboratory at Weill Cornell Medicine. Messenger RNA was prepared using TruSeq Stranded mRNA Sample Library Preparation kit (Illumina), according to the manufacturer’s instructions. The normalized libraries were pooled and sequenced on Illumina Novaseq 6000 sequencer with pair-end 50 cycles.The sequencing libraries sequenced with paired-end 50 bps on NovaSeq6000 sequencer.

The raw sequencing reads in BCL format were processed through bcl2fastq 2.19 (Illumina) for FASTQ conversion and demultiplexing. After trimming the adaptors with cutadapt (version1.18) (https://cutadapt.readthedocs.io/en/v1.18/), RNA reads were aligned and mapped to the GRCh38 human reference genome plus SARS-CoV-2 genome (MN985325.1) by STAR (Version2.5.2) (https://github.com/alexdobin/STAR)^45^, and transcriptome reconstruction was performed by Cufflinks (Version 2.1.1) (http://cole-trapnell-lab.github.io/cufflinks/). The abundance of transcripts was measured with Cufflinks in Fragments Per Kilobase of exon model per Million mapped reads (FPKM)^46,47^. Raw read counts per gene were extracted using HTSeq-count (Version0.11.2)^48^. Differential gene expression analysis was performed by DESeq2^49^. Principal component analysis was performed using Log_2_ FPKM values with R ggplot2 package. Heatmaps were generated using R gplots package. GO term and KEGG enrichment was analyzed using R clusterProfiler package^50^ and bar plots were generated using GraphPad Prism software. Gene Set Enrichment Analysis was performed by GSEA software (version 4.0.3)^51,52^.

### Single cell sequencing of hPSC-derived lung cells

Single-cell capture, reverse transcription, cell lysis, and library preparation was performed using the Single Cell 3′ version 3 kit and chip according to the manufacturer’s protocol (10× Genomics). Single-cell suspensions were generated by dissociating the cultured RUES2 cells with 0.05% Trypsin/0.02% EDTA for 10–15 min, followed with passing through 40 µM strainer. The single cell suspension was achieved through sorting the dissociated cells in flow cytometry singlets. Cell count was adjusted to 1000–2000 cells per ul to target an estimated capture of 8000 cells. Six input wells were used. Sequencing was performed on NovaSeq6000 with setting 28 for read 1 and 91 for read 2. The sequencing data were primarily analyzed by CellRanger pipeline v3.0.2 (10× Genomics). In particular, raw fastq data were generated by CellRanger *mkfastq*; A custom reference genome was built by integrating the virus and luciferase sequences into the 10× pre-built human reference (GRCh38 v3.0.0) using CellRanger *mkref*. Alignment of the raw reads to the custom reference genome, removing duplicated transcripts using the unique molecular identifiers (UMIs) and assignment to single cells was performed using CellRanger *count*.

Briefly, we used cells Seurat 3.1.4 R package for data analysis and visualization^53^. The Seurat object is required at least 200 and at most 6000 unique molecular identifiers (UMIs), genes detected (UMI count > 0) in less than two cells were removed. In addition, cells were excluded if more than 10% of sequences mapped to mitochondrial genes. In total, 5080 cells from the sample passed these filters for quality.

Following the package suggestions, we used a linear model to mitigate the variation stemming from the number of detected unique molecules per cell. The differentially expressed genes were found by *vst* method and the top 3000 differentially expressed genes were selected for PCA analysis. We used an elbow plot to determine the number of PCs. 20 PCs were used for each group of cells. Clustering resolution was set at 0.2. For co-culture analysis, Macrophages and lung cells were re-clustered and re-analyzed, respectively. Macrophages were integrated using the first 20 dimensions of PCs and clustering resolution was set at 0.1. UMAP plots, heatmaps, violin plots and dot plots were generated by the Seurat toolkit *FeaturePlot*, *DoHeatmap*, *VlnPlot* amd *DotPlot* functions, respectively. Cell types were determined using a combination of marker genes identified from the literature and the web-based tool Topp CellAtlas (https://toppgene.cchmc.org/).

### Quantification and statistical analysis

Sample sizes for all figures and tables were estimated based on our previous studies^19–21,54^. For each set of experiments, samples were prepared for all experimental arms at the same time. All statistical tests are two-sided. No adjustments were made for multiple comparisons. The relevant investigators (KZ, ZZ, FD, and LG) were blinded to experimental allocations among different experimental arms for all experiments. *N* = 3 independent biological replicates were used for all experiments unless otherwise indicated. n.s. indicates a non-significant difference. For all parametric and non-parametric tests, variances were similar between groups being compared. For comparison between experimental and control groups at a specific time point or tissue site, two-sided Student *t* tests, one-way ANOVA with Tukey’s multiple comparison test were used. All cells (RUES2, H1, hiPSC, HEK293T, THP-1, A549, Calu-3) were purchased from ATCC or WiCell in the past 2 years and were negative for mycoplasma. The hPSC lines were regularly checked for chromosome abnormalities and maintained with normal chromosome numbers.

### Reporting summary

Further information on research design is available in the Nature Research Reporting Summary linked to this article.

## Supplementary information

Supplementary Information

Reporting Summary

## Data Availability

The scRNA-seq data (hPSC-derived lung cells, co-culture of macrophage and lung cells derived from hPSC) are available from the GEO repository database with primary accession number GSE162996. Bulk RNA-seq data (macrophages derived from hPSC, macrophages derived from hPSC in SARS-CoV-2 infection) are accessible through GEO Series accession number GSE160631 (https://www.ncbi.nlm.nih.gov/geo/query/acc.cgi). Data that support the findings of this study have been included within the paper and the Supplementary Information file. Source data are provided with this paper.

## Source data

Source Data

## Code availability

All the codes and software used in the data analysis are available in Source Data file which are provided with this paper.

## References

[1] Guan W (2020). Clinical characteristics of coronavirus disease 2019 in China. N. Engl. J. Med..

[2] Mehta P (2020). COVID-19: consider cytokine storm syndromes and immunosuppression. Lancet.

[3] Wang D (2020). Clinical characteristics of 138 hospitalized patients with 2019 novel coronavirus–infected pneumonia in Wuhan, China. JAMA.

[4] Allard B, Panariti A, Martin JG (2018). Alveolar macrophages in the resolution of inflammation, tissue repair, and tolerance to infection. Front. Immunol..

[5] Xu Z (2020). Pathological findings of COVID-19 associated with acute respiratory distress syndrome. Lancet Resp. Med..

[6] Liao M (2020). Single-cell landscape of bronchoalveolar immune cells in patients with COVID-19. Nat. Med..

[7] Merad M, Martin JC (2020). Pathological inflammation in patients with COVID-19: a key role for monocytes and macrophages. Nat. Rev. Immunol..

[8] Wynn TA, Chawla A, Pollard JW (2013). Macrophage biology in development, homeostasis and disease. Nature.

[9] Gomez Perdiguero E (2015). Tissue-resident macrophages originate from yolk-sac-derived erythro-myeloid progenitors. Nature.

[10] Bian Z (2020). Deciphering human macrophage development at single-cell resolution. Nature.

[11] Shapouri‐Moghaddam A (2018). Macrophage plasticity, polarization, and function in health and disease. J. Cell Physiol..

[12] Mills CD (2015). Anatomy of a discovery: m1 and m2 macrophages. Front. Immunol..

[13] Murray PJ (2017). Macrophage polarization. Annu. Rev. Physiol..

[14] Jobe O (2017). Human primary macrophages derived in vitro from circulating monocytes comprise adherent and non-adherent subsets with differential expression of Siglec-1 and CD4 and permissiveness to HIV-1 infection. Front. Immunol..

[15] Zhang J (2021). Pyroptotic macrophages stimulate the SARS-CoV-2-associated cytokine storm. Cell Mol. Immunol..

[16] Hurley K (2020). Reconstructed single-cell fate trajectories define lineage plasticity windows during differentiation of human PSC-derived distal lung progenitors. Cell Stem Cell.

[17] Mou H (2012). Generation of multipotent lung and airway progenitors from mouse ESCs and patient-specific cystic fibrosis iPSCs. Cell Stem Cell.

[18] Dye BR (2015). In vitro generation of human pluripotent stem cell derived lung organoids. elife.

[19] Huang SX (2015). The in vitro generation of lung and airway progenitor cells from human pluripotent stem cells. Nat. Protoc..

[20] Huang SX (2014). Efficient generation of lung and airway epithelial cells from human pluripotent stem cells. Nat. Biotechnol..

[21] Chen HJ (2019). Generation of pulmonary neuroendocrine cells and SCLC-like tumors from human embryonic stem cells. J. Exp. Med..

[22] Travaglini KJ (2020). A molecular cell atlas of the human lung from single-cell RNA sequencing. Nature.

[23] Hoffmann M (2020). SARS-CoV-2 cell entry depends on ACE2 and TMPRSS2 and is blocked by a clinically proven protease inhibitor. Cell.

[24] Ziegler CG (2020). SARS-CoV-2 receptor ACE2 is an interferon-stimulated gene in human airway epithelial cells and is enriched in specific cell subsets across tissues. Cell.

[25] Duan F (2018). Biphasic modulation of insulin signaling enables highly efficient hematopoietic differentiation from human pluripotent stem cells. Stem Cell Res. Ther..

[26] Nico (2015). Large-scale hematopoietic differentiation of human induced pluripotent stem cells provides granulocytes or macrophages for cell replacement therapies. Stem Cell Rep..

[27] Lang J (2018). An hPSC-derived tissue-resident macrophage model reveals differential responses of macrophages to ZIKV and DENV infection. Stem Cell Rep..

[28] Xu C (2019). Differentiation and Functional Comparison of Monocytes and Macrophages from hiPSCs with Peripheral Blood Derivatives. Stem Cell Rep..

[29] Buchrieser J, James W (2017). Human Induced Pluripotent Stem Cell-Derived Macrophages Share Ontogeny with MYB-Independent Tissue-Resident Macrophages. Stem Cell Rep..

[30] Lopez-Yrigoyen M (2018). A human iPSC line capable of differentiating into functional macrophages expressing ZsGreen: a tool for the study and in vivo tracking of therapeutic cells. Philos. T. R. Soc. B.

[31] Zhang H (2015). Functional analysis and transcriptomic profiling of iPSC-derived macrophages and their application in modeling mendelian disease. Circ. Res..

[32] Li W (2003). Angiotensin-converting enzyme 2 is a functional receptor for the SARS coronavirus. Nature.

[33] Han Y (2021). Identification of SARS-CoV-2 inhibitors using lung and colonic organoids. Nature.

[34] Conti P (2020). Induction of pro-inflammatory cytokines (IL-1 and IL-6) and lung inflammation by COVID-19: anti-inflammatory strategies. J. Biol. Reg. Homeos.

[35] Chen H (2020). JAK1/2 pathway inhibition suppresses M2 polarization and overcomes resistance of myeloma to lenalidomide by reducing TRIB1, MUC1, CD44, CXCL12, and CXCR4 expression. Br. J. Haematol..

[36] Tay MZ, Poh CM, Rénia L, Macary PA, Ng LF (2020). The trinity of COVID-19: immunity, inflammation and intervention. Nat. Rev. Immunol..

[37] McCray PB (2007). Lethal infection of K18-hACE2 mice infected with severe acute respiratory syndrome coronavirus. J. Virol..

[38] Kim YI (2020). Infection and rapid transmission of SARS-CoV-2 in Ferrets. Cell Host Microbe.

[39] Huang C (2020). Clinical features of patients infected with 2019 novel coronavirus in Wuhan, China. Lancet.

[40] Morais-Almeida M (2020). COVID-19, asthma, and biological therapies: What we need to know. World Allergy Organ.

[41] Luo, W. et al. Histopathologic findings in the explant lungs of a patient with COVID-19 treated with bilateral orthotopic lung transplant. *Transplantation* **104**, e329–e331 (2020). 10.1097/TP.0000000000003412 33122591

[42] National Health CommissionNational Administration of Traditional Chinese Medicine (2020). Diagnosis and treatment protocol for novel coronavirus pneumonia (Trial Version 7). Chin. Med. J..

[43] Plasschaert LW (2018). A single-cell atlas of the airway epithelium reveals the CFTR-rich pulmonary ionocyte. Nature.

[44] Blanco-Melo D (2020). Imbalanced host response to SARS-CoV-2 drives development of COVID-19. Cell.

[45] Dobin A (2013). STAR: ultrafast universal RNA-seq aligner. Bioinformatics.

[46] Trapnell C (2010). Transcript assembly and quantification by RNA-Seq reveals unannotated transcripts and isoform switching during cell differentiation. Nat. Biotechnol..

[47] Trapnell C (2013). Differential analysis of gene regulation at transcript resolution with RNA-seq. Nat. Biotechnol..

[48] Anders S, Pyl PT, Huber W (2015). HTSeq—a Python framework to work with high-throughput sequencing data. Bioinformatics.

[49] Love MI, Huber W, Anders S (2014). Moderated estimation of fold change and dispersion for RNA-seq data with DESeq2. Genome Biol..

[50] Yu G, Wang LG, Han Y, He Q (2012). Y. clusterProfiler: an R package for comparing biological themes among gene clusters. Omics: J. Integr. Biol..

[51] Subramanian A (2005). Gene set enrichment analysis: a knowledge-based approach for interpreting genome-wide expression profiles. Proc. Natl Acad. Sci. USA.

[52] Mootha VK (2003). PGC-1α-responsive genes involved in oxidative phosphorylation are coordinately downregulated in human diabetes. Nat. Genet..

[53] Butler A, Hoffman P, Smibert P, Papalexi E, Satija R (2018). Integrating single-cell transcriptomic data across different conditions, technologies, and species. Nat. Biotechnol..

[54] Han Y (2021). Identification of SARS-CoV-2 inhibitors using lung and colonic organoids. Nature.

