# Supplementary material for: Differential effects of macrophage subtypes on SARS-CoV-2 infection in a human pluripotent stem cell-derived model
Source: Nat Commun. 2022 Apr 19;13:2028. doi: 10.1038/s41467-022-29731-5 (PMC9018716; doi:10.1038/s41467-022-29731-5)
Supplement: Supplementary file 1 — Supplementary Information [file 41467_2022_29731_MOESM1_ESM.pdf]

# Differential Effects of Macrophage Subtypes on SARS-CoV-2 Infection in A Human Pluripotent Stem Cell-Derived Model

## Supplementary Table 1 and Figures 1-14

**Authors:** Qizhou Lian[1,2#]*, Kui Zhang[3, 4#], Zhao Zhang[2#], Fuyu Duan[1#], Liyan Guo[1#], Weiren Luo[5], Bobo Wing-Yee Mok[6], Abhimanyu Thakur[3,4], Xiaoshan Ke[3, 4], Pedram Motallebnejad [3,4], Vlad Nicolaescu[7], Jonathan Chen[8], Chui Yan Ma[1], Xiaoya Zhou[2], Shuo Han[9] ,Teng Han[10], Wei Zhang[11], Adrian Y Tan[11], Tuo Zhang[11], Xing Wang[11], Dong Xu[11], Jenny Xiang[11], Aimin Xu[12], Can Liao[1], Fang-Ping Huang[13], Ya-Wen Chen[14,15], Jie Na [16], Glenn Randall[7], Hung-fat Tse[2], Zhiwei Chen[17], Yin Chen[18]， Huanhuan Joyce Chen[3,4,*]

**Affiliations**

[1] Cord Blood Bank Center, Guangzhou Women and Children's Medical Center, Guangzhou Medical University, Guangzhou, China.

[2] HKUMed Laboratory of Cellular Therapeutics, and Department of Medicine, the University of Hong Kong, Hong Kong SAR, China

[3]The Pritzker School of Molecular Engineering, the University of Chicago, IL. 60637, USA.

[4] The Ben May Department for Cancer Research, the University of Chicago, IL. 60637, USA.

[5] Department of Pathology, The Second Affiliated Hospital of Southern University of Science and Technology, Shenzhen Third People's Hospital, National Clinical Research Centre for Infectious Diseases, Shenzhen, China.

[6]Department of Microbiology and State Key Laboratory for Emerging Infectious Diseases, Li Ka Shing Faculty of Medicine, The University of Hong Kong, Hong Kong, SAR, China.

[7] Microbiology and Immunology, Biosciences Division, the University of Chicago, IL. 60637, USA

[8] McCormick School of Engineering, Northwestern University, IL. USA

[9]School of Biomedical Sciences, Li Ka Shing Faculty of Medicine, The University of Hong Kong, Hong Kong, SAR, China

[10] Sandra and Edward Meyer Cancer Center, Department of Medicine, Weill Cornell Medicine, New York, 10021, USA

[11]Genomic Resource Core Facility, Weill Cornell Medicine, New York, NY 10065, USA.

[12]State Key Laboratory of Pharmaceutical Biotechnology, Li Ka Shing Faculty of Medicine, The University of Hong Kong, Hong Kong, SAR, China.

[13] Institute for Advanced Study (IAS), Shenzhen University, Shenzhen, China.

[14] Department of Medicine, Hastings Center for Pulmonary Research, Division of Pulmonary, Critical Care and Sleep Medicine, [15]Department of Stem Cell Biology and Regenerative Biology, Keck School of Medicine, University of Southern California, Los Angeles, CA 90089.

[16] School of Medicine, Tsinghua University, Beijing, China.

[17]AIDS institute and department of microbiology, State Key Laboratory of Emergent Infectious Disease, The University of Hong Kong, Hong Kong China.

[18] Department of Pharmacology and Toxicology, School of Pharmacy, University of Arizona, Tucson, AZ. USA

[#] **These authors contributed equally**

***Corresponding authors**
Correspondence to
Dr. Huanhuan Joyce Chen: joycechen@uchicago.edu; and Dr. Qizhou Lian: qzlian@hku.hk

**Supplementary Table 1. Key resources table**

| REAGENT or RESOURCE | SOURCE | IDENTIFIER | |
|---|---|---|---|
| **Antibodies** | | | |
| **Antibody** | **Sources** | **Catalog no.** | **Application & Dilution** |
| Mouse monoclonal Anti-CD68 | eBioscience | #14-0688-82 | IF 1:100 |
| Rabbit monoclonal Anti-CD68 | Abcam | #ab213363 | IF 1:100<br>IHC 1:500 |
| Rabbit polyclonal anti-CD80 | BOSTER | #A00196-1 | IF 1:100 |
| Mouse monoclonal Anti-CD163 | Abcam | #ab156769 | IF 1:100 |
| Rabbit monoclonal anti-CD163 | Abcam | #ab182422 | IF 1:100 |
| Mouse monoclonal anti-CD80-PE | miltenyi Biotec | #130-117-683 | IF 1:100 |
| Mouse monoclonal anti-CD206-PE | miltenyi Biotec | #130-095-220 | IF 1:100 |
| Rabbit polyclonal Anti-CD206 | Abcam | #ab64693 | IF 1:200 |
| Rabbit monoclonal Anti-FABP4 | Abcam | #ab92501 | IF 1:50 |
| Mouse monoclonal Anti-HLA-DR | eBioscience | #14-9956-80 | IF 1:100 |
| Rabbit polyclonal anti-IL-6 | Affinity | #DF6087 | IF 1:200 |
| Rabbit polyclonal Anti-IL-32 | Abcam | #ab37158 | IF 1:100 |
| Mouse monoclonal Anti-CCL2 | Abcam | #ab9858 | IF 1:100 |
| Rabbit monoclonal Anti-IL-1 beta | Abcam | #ab254360 | IF 1:100 |
| Goat polyclonal anti-ACE2 | R&D systems | #AF933 | FACS 5μl/ million cells |
| Mouse monoclonal anti-CD68-PE | Biolegend | #333807 | FACS 5μl/ million cells |
| Mouse monoclonal anti-CD11b, APC-conjugated | Biolegend | #301309 | FACS 5μl/ million cells |
| Mouse monoclonal anti-CD14, FITC-conjugated | Biolegend | #325603 | FACS 5μl/ million cells |
| FITC Mouse IgG1, κ Isotype Ctrl | Biolegend | #400107 | FACS 5μl/ million cells |
| PE Mouse IgG1, κ Isotype Ctrl | Biolegend | #400139 | FACS 5μl/ million cells |
| APC Mouse IgG1, κ Isotype Ctrl | Biolegend | #400120 | FACS 5μl/ million cells |
| Rabbit polyclonal Anti-NKX2.1 | Seven Hills Bioreagents | #WRAB-1231 | IF 1:500 |
| Goat polyclonal Anti-FOXA2 | Santa Cruz | #sc-6554 | IF 1:100 |

| | | | |
|---|---|---|---|
| Goat polyclonal Anti-SOX2 | Santa Cruz | #sc-17320 | IF 1:100 |
| Rabbit polyclona Anti-SP-B | Seven Hills Bioreagents | #WRAB-48604 | IF 1:500 |
| Rabbit polyclona Anti-Pro-SP-C | Seven Hills Bioreagents | #WRAB-9337 | IF 1:500 |
| Rabbit polyclona Anti-FOXJ1 | Sigma-Aldrich | #HPA005714-1 | IF 1:100 |
| Anti-SARS-CoV-2 Nucleocapsid Antibody | R&D systems | #MAB10474 | FACS 5µl/ million cells |
| SARS-CoV/SARS-CoV-2 Nucleocapsid Antibody | Sino Biological | #40143-MM05 | FACS 5µl/ million cells |
| Rabbit polyclonal Anti-LAMP2 | Santa Cruz | #sc-5571 | IF 1:100 |
| Donkey anti-Mouse IgG (H+L) Highly Cross-Adsorbed Secondary Antibody, Alexa Fluor 488 | Thermo Fisher Scientific | #A-21202 | IF 5 µg/mL |
| Donkey anti-Mouse IgG (H+L) Highly Cross-Adsorbed Secondary Antibody, Alexa Fluor 594 | Thermo Fisher Scientific | #A-21203 | IF 5 µg/mL |
| Donkey anti-Rabbit IgG (H+L) Secondary Antibody, Alexa Fluor 594 conjugate | Thermo Fisher Scientific | #A-21207 | IF 5 µg/mL |
| Goat anti-Rabbit IgG (H+L) Cross-Adsorbed Secondary Antibody, Alexa Fluor 488 | Thermo Fisher Scientific | #A-11008 | IF 5 µg/mL |
| Donkey anti-Rabbit IgG (H+L) Secondary Antibody, Alexa Fluor 647 conjugate | Thermo Fisher Scientific | #A-31573 | IF 5 µg/mL |
| Donkey anti-Mouse IgG (H+L) Secondary Antibody, Alexa Fluor 647 | Thermo Fisher Scientific | #A-31571 | IF 5 µg/mL |
| Donkey anti-Goat IgG (H+L) Cross-Adsorbed Secondary Antibody, Alexa Fluor 647 | Thermo Fisher Scientific | #A-21447 | IF 5 µg/mL |
| **Chemicals, Peptides, and Recombinant Proteins** | | | |
| Activin A | R&D Systems | #338-AC-500/CF | |
| Y-27632 | MedchemExpress | #HY-10583 | |
| Recombinant Human BMP-4 Protein | R & D Systems | #314-BP | |
| Recombinant Human bFGF | R&D Systems | #233-FB-500 | |
| Dorsomorphin dihydrochloride | R&D Systems | #3093/50 | |
| SB431542 | R&D Systems | #1614/50 | |
| IWP2 | R&D Systems | #3533/50 | |

| | | |
|---|---|---|
| CHIR99021 | Cayman Chemical | #13122 |
| Recombinant Human FGF-10 Protein | R&D Systems | #345-FG-250 |
| Recombinant Human KGF/FGF-7 Protein | R&D Systems | #251-KG-01M |
| Retinoic acid | Sigma-Aldrich | #R2625 |
| Dexamethasone | Sigma-Aldrich | #D4902 |
| 8-Bromo-cAMP | Sigma-Aldrich | #B5386 |
| IBMX | Sigma-Aldrich | #I5879 |
| Recombinant Human VEGF Protein | R&D Systems | #293-VE-500/CF |
| Recombinant Human IL-3 Protein | R&D Systems | #203-IL-050/CF |
| Recombinant Human M-CSF Protein | R&D Systems | #216-MC-025 |
| Recombinant Human IL-4 Protein | R&D Systems | #204-IL-050 |
| IFNγ | R&D Systems | #285-IF-100 |
| LPS | Sigma-Aldrich | #L4391-1MG |
| DAPI | Santa Cruz | #sc-3598 |
| Hoechst 33342 | Sigma-Aldrich | # B2261-100mg |
| Wright-Giemsa Stain | Sigma-Aldrich | #WG16-500ML |
| Latex beads, carboxylate-modified polystyrene | Sigma-Aldrich | #L4655 |
| Alexa Fluor™ 488 Phalloidin | Thermo Fisher Scientific | #A12379 |
| **Culture Medium** | | |
| F12 | Gibco Thermo Fisher | #31765035 |
| β-mercaptoethanol | Sigma Aldrich | #M3148 |
| Penicillin-Streptomycin (5,000 U/mL) | Gibco Thermo Fisher | #15070063 |
| MEM Non-Essential Amino Acids Solution (100X) | Gibco Thermo Fisher | #11140050 |
| IMDM | Gibco Thermo Fisher | #21056023 |
| GlutaMAX Supplement | Thermo Fisher Scientific | #35050079 |
| Accutase | Stemcell Technologies | #07920 |
| Matrigel | Corning | #354234 |
| Fibronectin (FN) | Thermo Fisher Scientific | #356008 |
| N2 supplement | Thermo Fisher Scientific | #17502-048 |
| B27 | Thermo Fisher Scientific | #12587-010 |
| DMEM/F12 | Thermo Fisher Scientific | #10565-018 |

| | | |
|---|---|---|
| Knockout serum replacement (KOSR) | Thermo Fisher Scientific | #10828-028 |
| FBS | Gibco Thermo Fisher | #10099141C |
| Monothioglycerol | Sigma Aldrich | #M6145 |
| Ascorbic acid | Sigma Aldrich | #A4403 |
| Bovine serum albumin (BSA) | Sigma Aldrich | #A9418 |
| **Experimental Models: Cell Lines** | | |
| hESC line H1 | WiCell | #WAe001-A |
| hESC line-RUES2 | WiCell | #RUESe002-A |
| hiPS line (IMR90-1) | WiCell | #WISCi004-A |
| HEK293T | ATCC | #CRL-11268 |
| Vero E6 | ATCC | #CRL-1586 |
| THP-1 | ATCC | #TIB-202 |
| Mouse embryonic fibroblasts | Global Stem | #GSC-6001G |
| A549 | ATCC | #CCL-185 |
| Calu-3 | ATCC | #HTB-55 |
| **Software and Algorithms** | | |
| Cell Ranger | 10X Genomics | https://support.10xgenomics.com/single-cell-gene-expression/software/overview/welcome |
| Scran | doi: 10.12688/f1000research.9501.2. | https://bioconductor.org/packages/release/bioc/html/scran.html |
| Rstudio | Rstudio | https://rstudio.com |
| Seurat R package v3.1.4 | https://doi.org/10.1038/nbt.4096 | https://satijalab.org/seurat/ |
| Adobe illustrator CC2017 | Adobe | https://www.adobe.com/products/illustrator.html |
| Graphpad Prism 8.0 | Graphpad software | https://www.graphpad.com |
| FlowJo v x.0.7 | BD Biosciences | https://www.flowjo.com/ |
| ToppCell Atlas | Toppgene | https://toppgene.cchmc.org/ |

**Supplementary Fig. 1: Macrophages subpopulations in non-COVID-19 vs. COVID-19 patients**

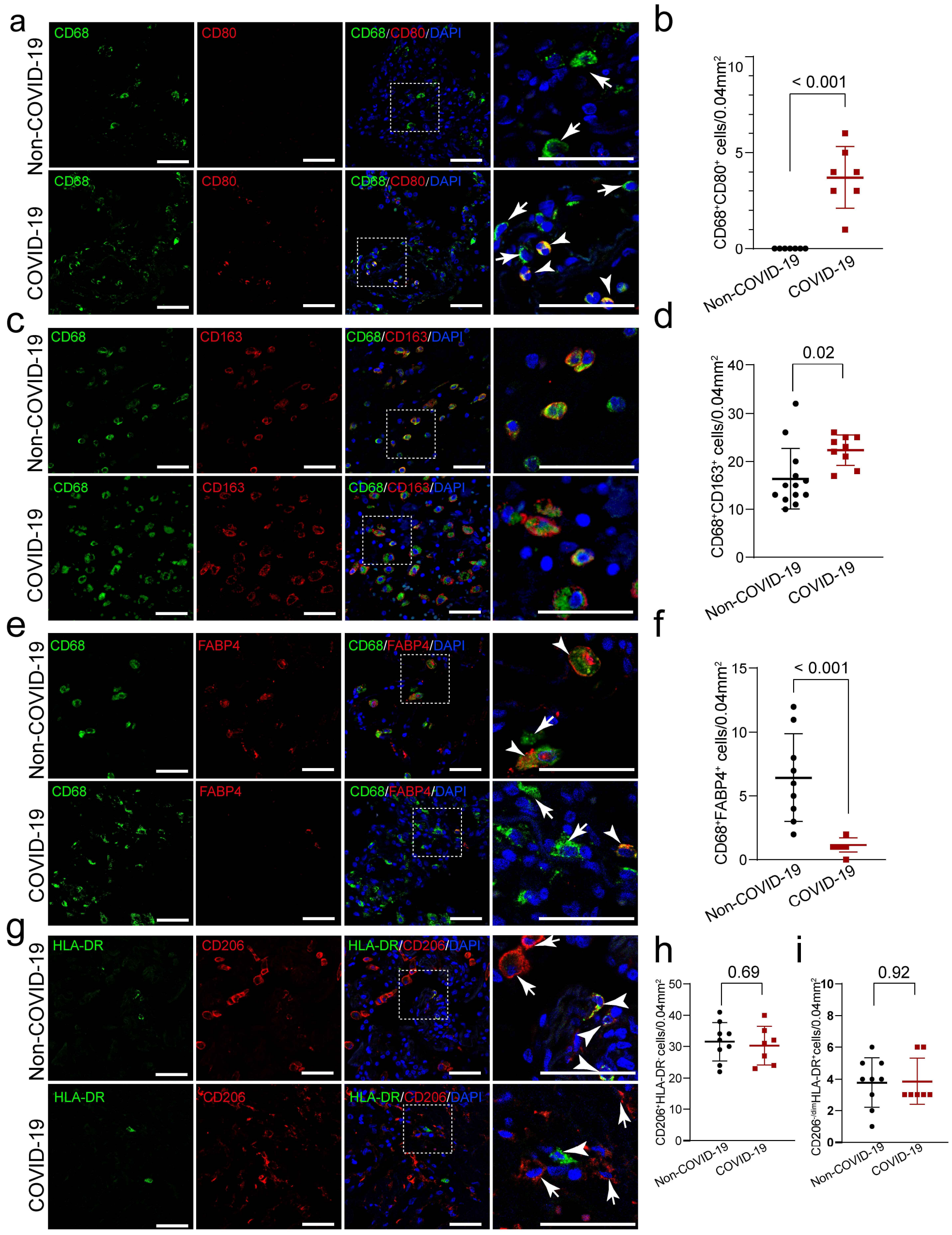


**Supplementary Fig. 1: Macrophages subpopulations in non-COVID-19 vs. COVID-19 patients**

a. IF staining of non-COVID-19 or COVID-19 distal lung tissues using antibodies against CD68 and CD80. Scale bar: 50 μm.

b. Quantification of $CD68^+CD80^+$ macrophages in Non-COVID-19 or COVID-19 distal lung tissues. Statistically significant differences are calculated using an unpaired two-tailed unpaired Student’s t-test.

c. IF staining of non-COVID-19 or COVID-19 distal lung tissues using antibodies against CD68 and CD163. Scale bar: 50 μm.

d. Quantification of $CD68^+CD163^+$ macrophages in Non-COVID-19 or COVID-19 distal lung tissues. Statistically significant differences are calculated using an unpaired two-tailed unpaired Student’s t-test.

e. IF staining of Non-COVID-19 or COVID-19 distal lung tissues using antibodies against CD68 and FABP4. Scale bar: 50 μm.

f. Quantification of $CD68^+FABP4^+$ macrophages in Non-COVID-19 or COVID-19 distal lung tissues. Statistically significant differences are calculated using an unpaired two-tailed unpaired Student’s t-test.

g. IF staining of non-COVID-19 or COVID-19 distal lung tissues using antibodies against CD206 and HLA-DR. Scale bar: 50 μm.

h. Quantification of $CD206^{+}HLA^{-}$ alveolar macrophages in Non-COVID-19 or COVID-19 distal lung tissues. Statistically significant differences are calculated using an unpaired two-tailed unpaired Student's t-test.

i. Quantification of $CD206^{-/dim}HLA^{+}$ interstitial macrophages in Non-COVID-19 or COVID-19 distal lung tissues. The data show mean ± standard deviation. Statistically significant differences are calculated using an unpaired two-tailed unpaired Student's t-test. n = 3 independent experiments.

**Supplementary Fig. 2: Macrophages subpopulations in non-COVID-19 vs. COVID-19 patients**

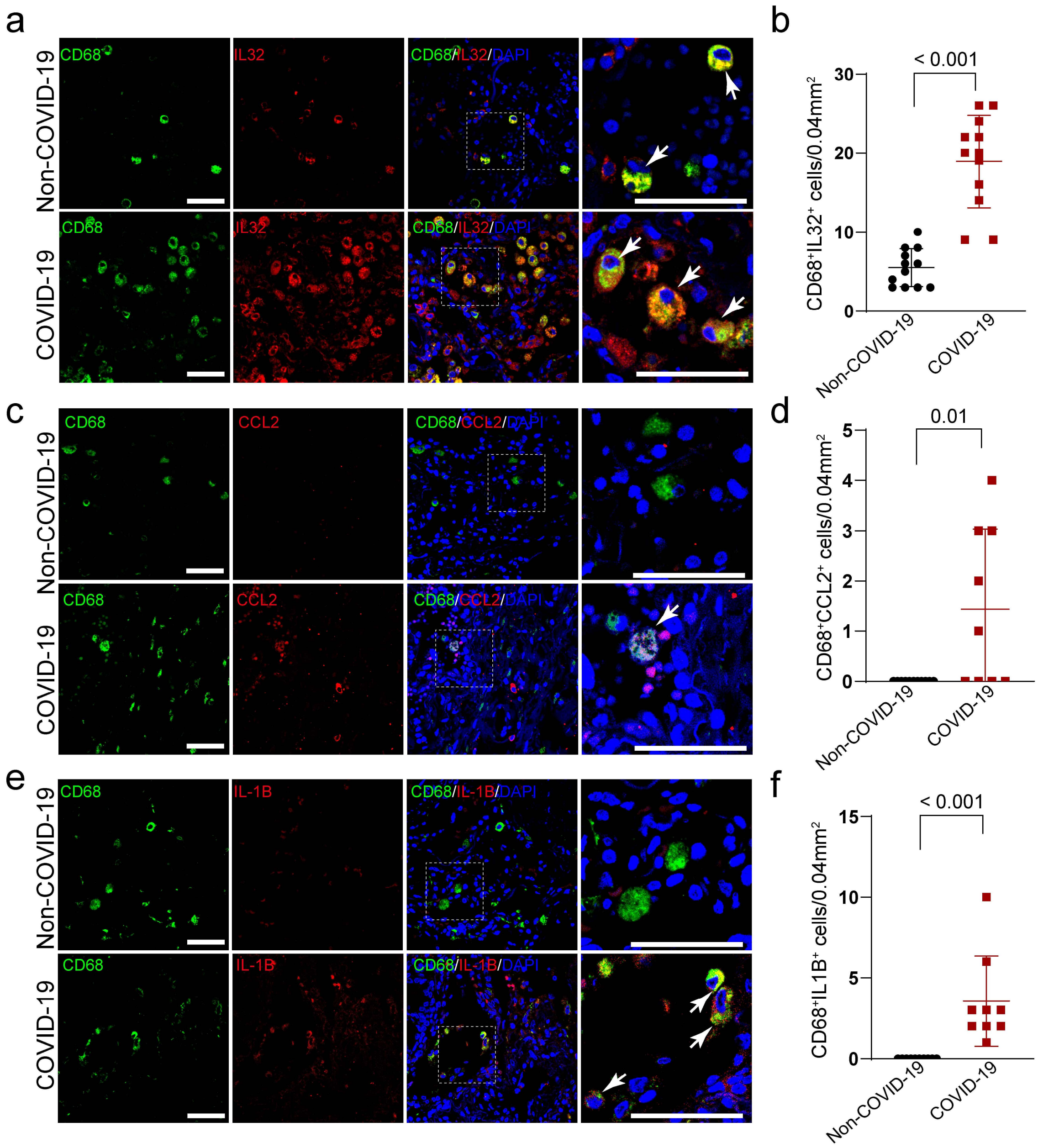


**Supplementary Fig. 2: Macrophages subpopulations in non-COVID-19 vs. COVID-19 patients**

a. IF staining of non-COVID-19 or COVID-19 distal lung tissues using antibodies against CD68 and IL-32. Scale bar: 50 μm.

b. Quantification of CD68+IL-32+ macrophages in Non-COVID-19 or COVID-19 distal lung tissues. Statistically significant differences are calculated using an unpaired two-tailed unpaired Student's t-test.

c. IF staining of non-COVID-19 or COVID-19 distal lung tissues using antibodies against CD68 and CCL2. Scale bar: 50 μm.

d. Quantification of CD68+CCL2+ macrophages in Non-COVID-19 or COVID-19 distal lung tissues. Statistically significant differences are calculated using an unpaired two-tailed unpaired Student's t-test.

e. IF staining of non-COVID-19 or COVID-19 distal lung tissues using antibodies against CD68 and IL-1B. Scale bar: 50 μm.
f. Quantification of CD68+IL-1B+ macrophages in Non-COVID-19 or COVID-19 distal lung tissues. Statistically significant differences are calculated using an unpaired two-tailed unpaired Student's t-test. n = 3 independent experiments. The data show mean ± standard deviation.

**Supplementary Fig. 3: Generation of lung cells by directed differentiation of hPSC**

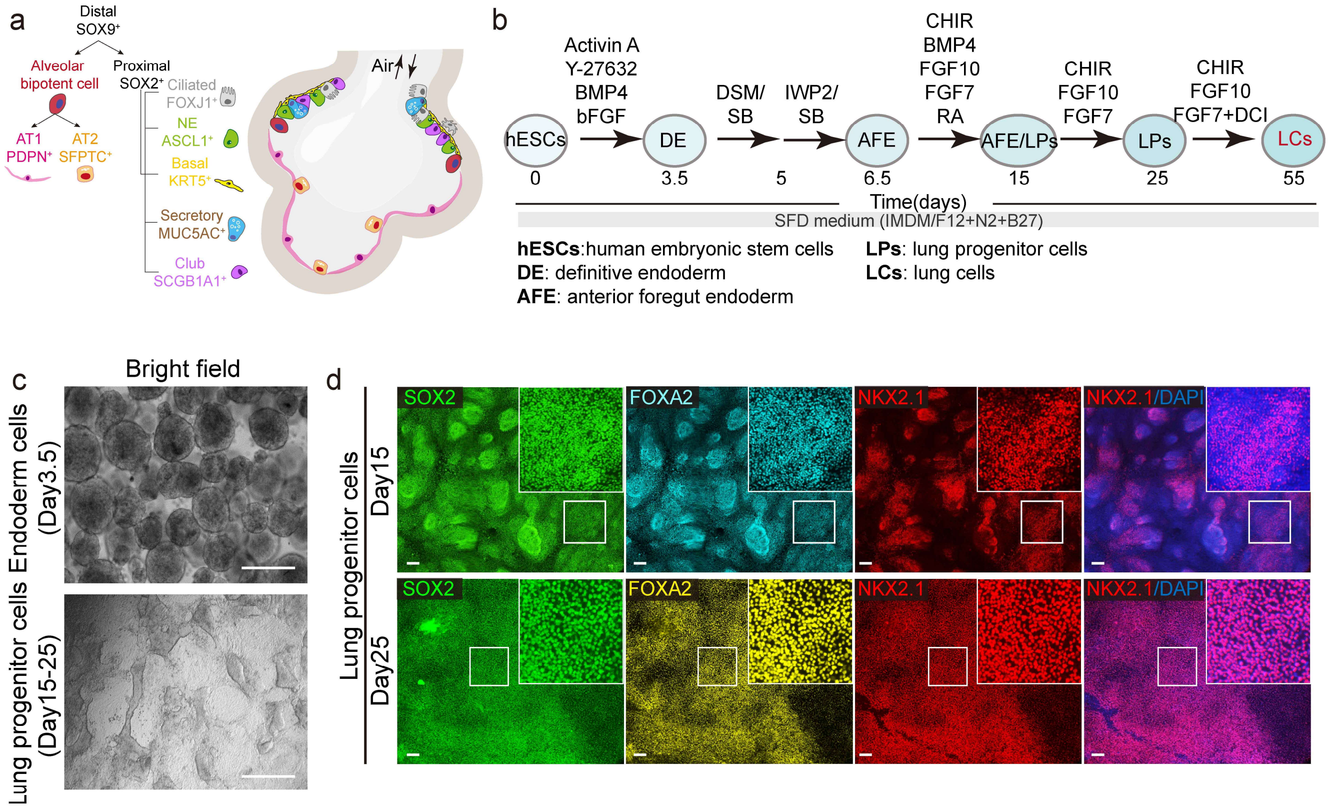


**Supplementary Fig. 3: Generation of lung cells by directed differentiation of hPSC**

a. Scheme of distal lung structure and gene signatures of lung lineage cells.

b. Scheme of directed differentiation of hPSC to lung organoids.

c, d Representative bright-field and IF images of the hPSC-derived cell cultures at day 3.5, 15 -25; indicating the cells that are triple-positive for SOX2, FOXA2, and NKX2.1 at anterior foregut endoderm/lung progenitor stage or lung progenitor stage. Scale bars=100 µm. Images are representative of three independent experiments.

**Supplementary Fig. 4: ACE2 expression in hPSC-derived lung cells**

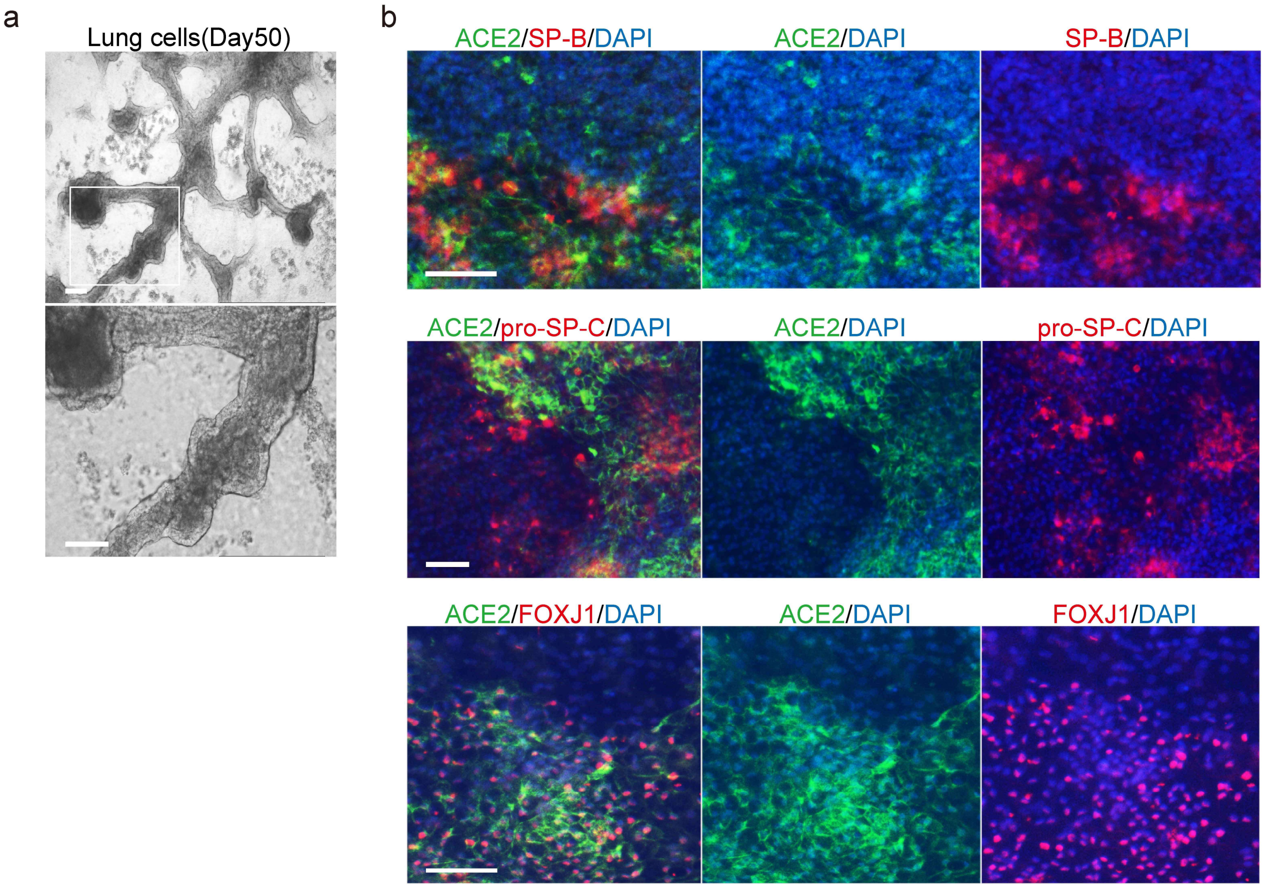


**Supplementary Fig. 4: ACE2 expression in hPSC-derived lung cells**

a. Representative bright-field images of the hPSC-derived lung cells at day 50 in the Matrigel-coated 2-D culture. Scale bars=200 μm. Images are representative of three independent experiments.

b. Expression of ACE2 was detected by immunostaining in hPSC-derived lung cells at day 50, in SP-B+ or Pro-SP-C+ AT2 cells, and FOXJ1+ ciliated cells. Scale bars= 100 μm. Images are representative of three independent experiments.

## Supplementary Fig. 5: Characterization of hPSC-derived lung cells by scRNA seq

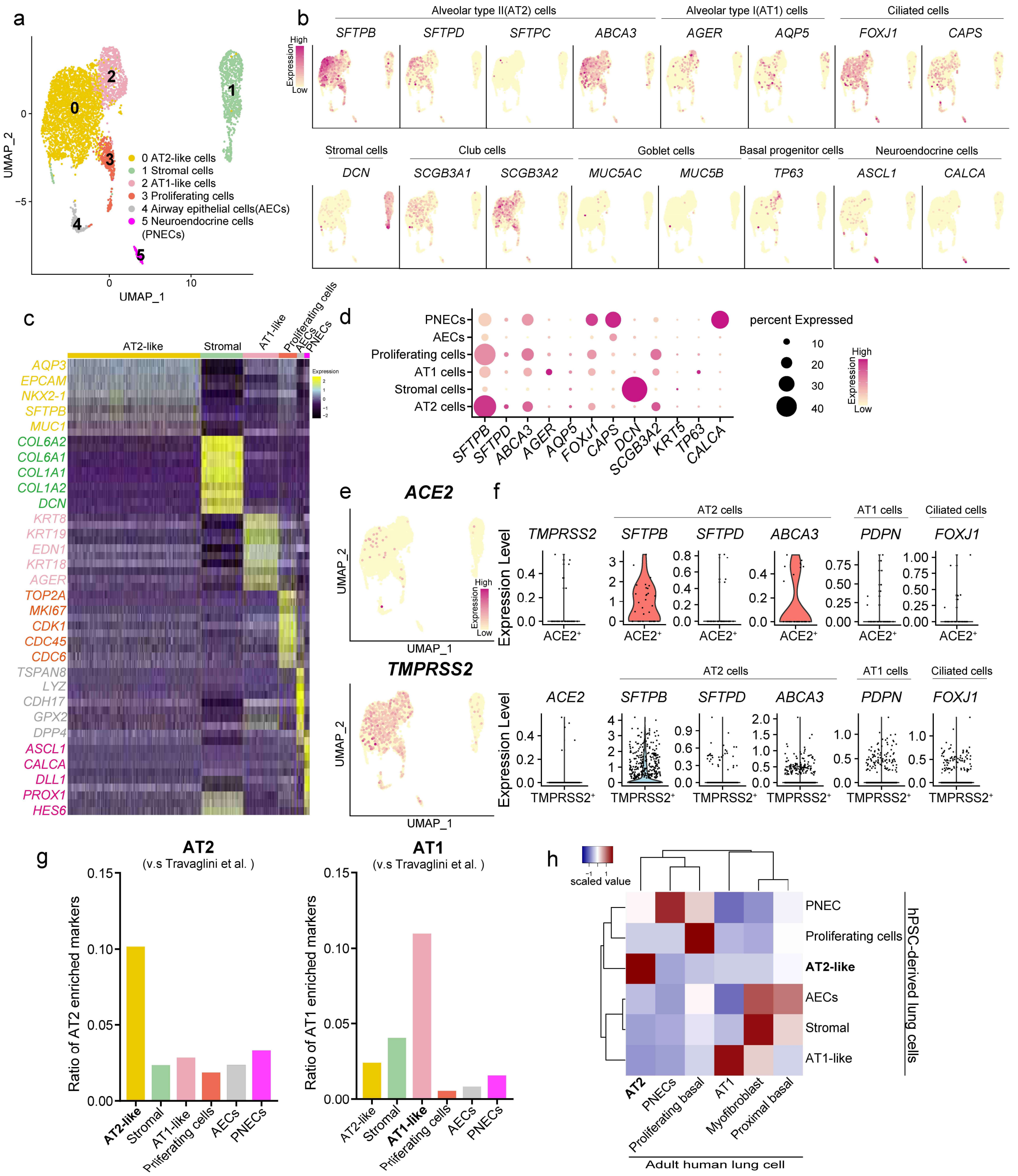


## Supplementary Fig. 5: Characterization of hPSC-derived lung cells by scRNA seq

a. UMAP hPSC-derived lung cells at day 50, colored and annotated with cluster 0-5. AT1, alveolar epithelial type 1 cells, AT2 cells, alveolar epithelial type 2 cells.

b. Putative lung cell-fate related markers differentially expressed in each cluster in UMAPs. Relative expression of each marker gene ranges from low (light yellow) to high (pink) as indicated. Individual cell positive for lung cell markers are donated by red dots.

c. Heatmap examination of each cluster for top differentially expressed genes.

d. Dot plot of gene signatures known for lung cell types in 5 clusters of hPSC-derived lung cell culture. Relative expression levels of each gene range from low (yellow) to high (red) as indicated. Percentages of cells with differential gene expression are indicated by the size of circles.

e, f Expression of ACE2 and TMPRSS2, in each cluster in UMAPs. The violin plot shows the expression level (log2(UMI+1)) of the indicated gene in each cluster. ACE2 and TMPRSS2 enriched in AT2, AT1, and ciliated cells.

g. Enrichment analysis of hPSC-derived lung cells using 205 and 1087 genes highly expressed in adult human AT2 cells and AT1 cells, respectively.

h. Correlation analysis of genes with cell fates in hPSC-derived lung cells and adult human lung cells. Relative correlation level of each cluster ranges from low (blue) to high (dark red), as indicated.

## Supplementary Fig. 6: Generation and characterization of macrophages by directed differentiation of hPSC

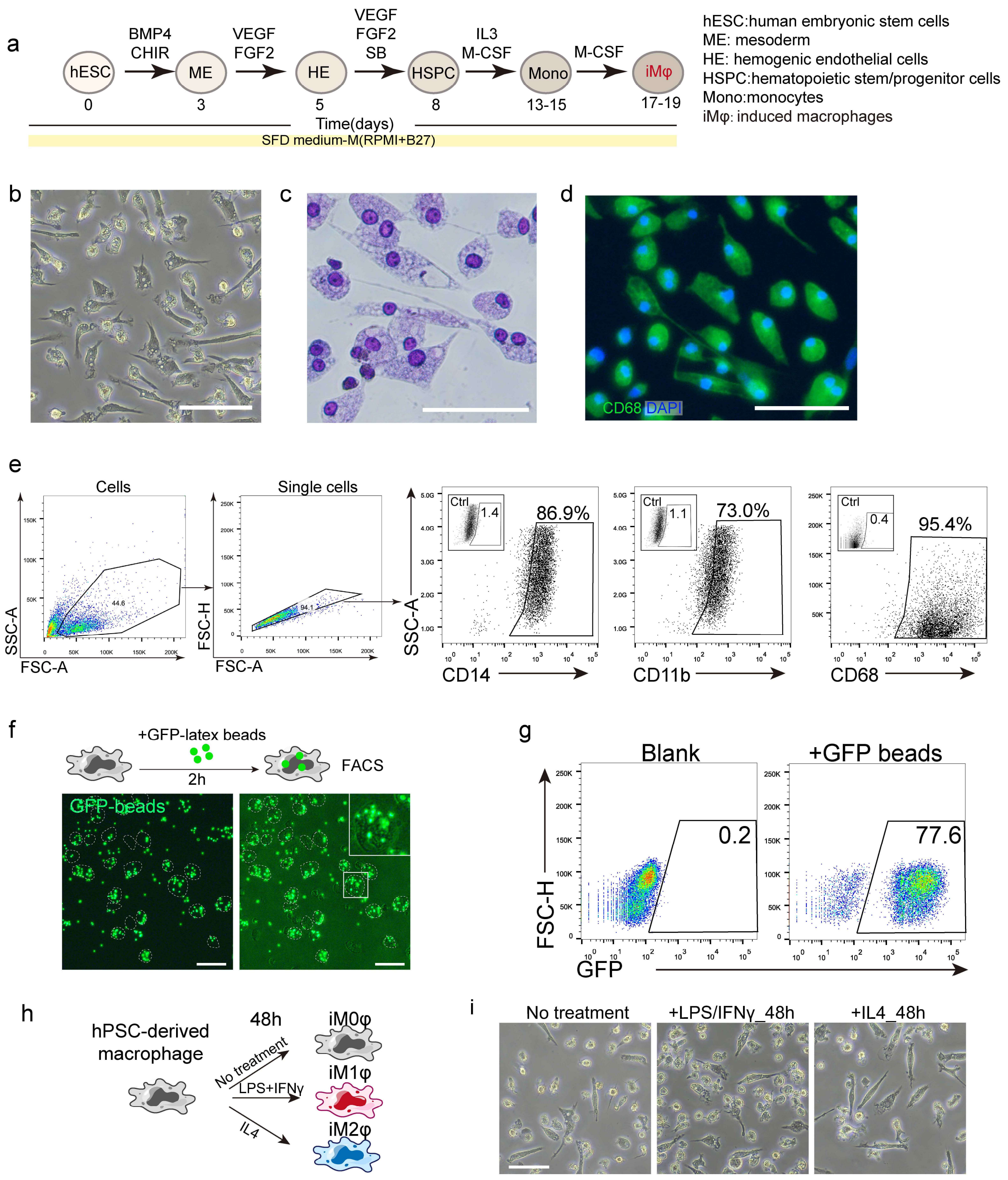


## Supplementary Fig. 6: Generation and characterization of macrophages by directed differentiation of hPSC

a. Scheme of directed differentiation of hPSC to macrophages.
b. Representative bright image on hPSC-derived macrophages in culture. Scale bars = 50µm. Images are representative of three independent experiments.
c. Giemsa staining of hPSC-derived macrophages.Scale bars = 50µm. Images are representative of three independent experiments.
d. Expression of CD68 was detected by immunostaining at day16. Scale bars= 50 µm. Images are representative of three independent experiments.

e. Flow cytometry characterized monocyte/macrophage markers CD14, CD11b and CD68 in hPSC-derived macrophages.
f. Phagocytosis test of iMAC using GFP-labeled latex beads(1um). iMACs are circled with dashed lines,scale bars = 50 μm. Images are representative of three independent experiments.
g. FACS analysis of engulfed GFP-beads by iMACs.
h. Schematic of the polariztion on hPSC-derived macrophages.
i. Morphology of the hPSC-derived macrophages at 48h-post treatment by LPS+IFNγ or IL-4. Scale bars= 50 μm. Images are representative of three independent experiments.

**Supplementary Fig. 7: Characterization of iM1φ and iM2φ cells polarized from hPSC-derived macrophages**

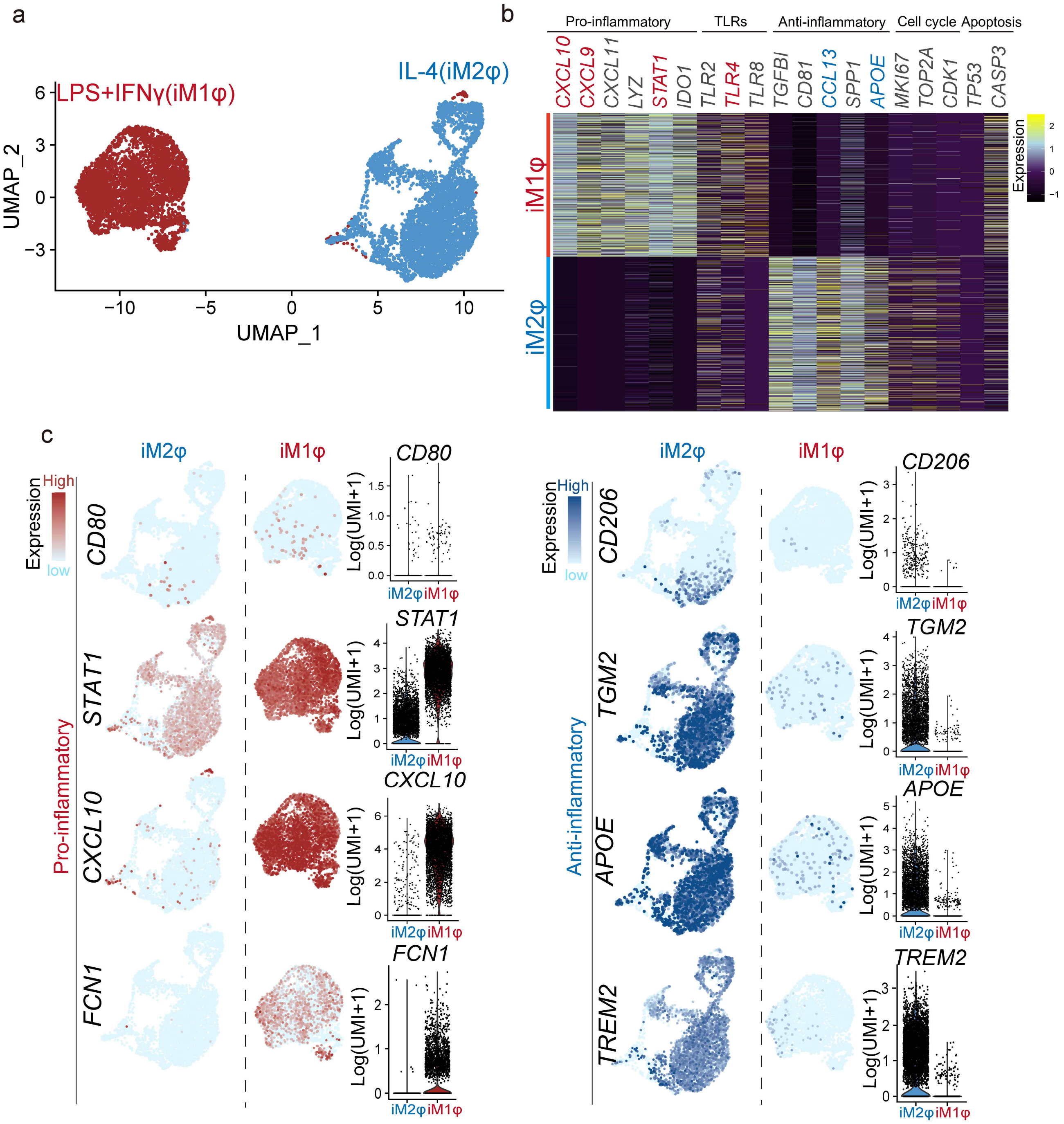


**Supplementary Fig. 7: Characterization of iM1φ and iM2φ cells polarized from hPSC-derived macrophages.** scRNA seq was performed on the co-cultures (iLung and iM1φ co-culture; iLung and iM2φ co-culture), (See Figure S8 on iLung)

a. UMAP of iM1φs(red) and iM2φs (blue) polarized by LPS/IFNγ and IL-4, respectively.

b. Heatmap presenting top differential expression genes related to pro- or anti-inflammatory factors, Toll like receptors (TLRs), cell cycle regulation, and apoptosis, in iM2φ or iM1φ cells.

c. A set of pro-or anti-inflammatory factors, or cell-fate related markers differentially expressed in the cluster of iM1φs or iM2φs in UMAPs. Relative expression of each marker gene ranges from low (light blue) to high (red in M1 and dark blue in M2) as indicated. The violin plot shows the expression level (log2(UMI+1)) of the indicated gene in each cluster.

**Supplementary Fig. 8: GO and KEGG analysis of the genes or pathways that were upregulated or enriched in iM1φ or iM2φ compared with iM0φ**

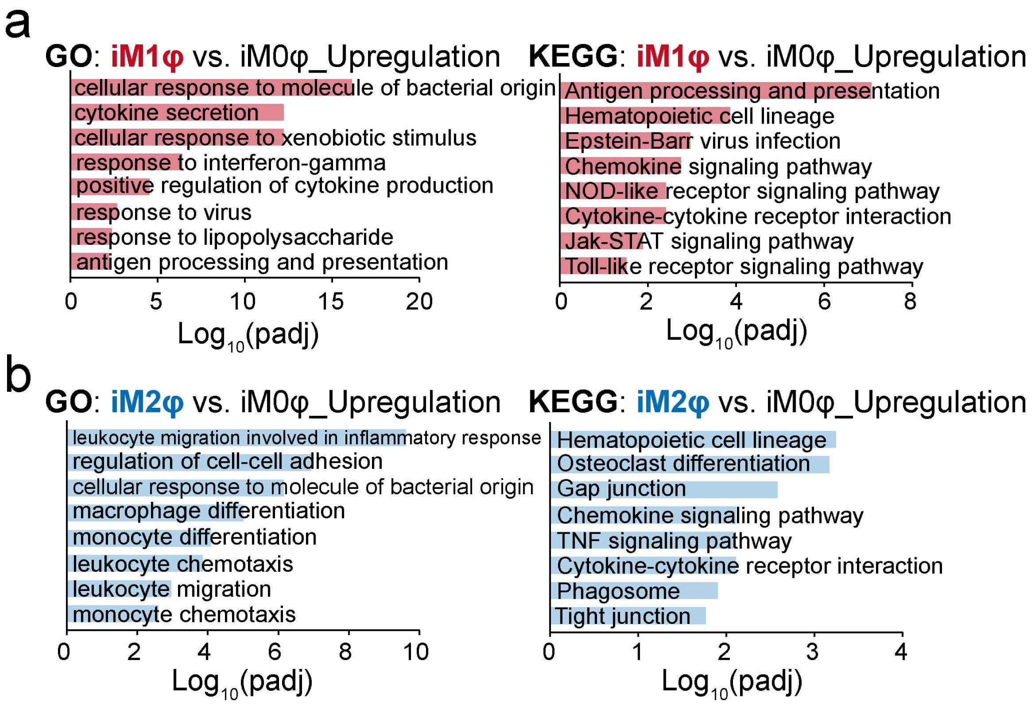


**Supplementary Fig. 8: GO and KEGG analysis of the genes or pathways that were upregulated or enriched in iM1φ or iM2φ compared with iM0φ.**

a. GO and KEGG analysis of the genes or pathways that were upregulated or enriched in iM1φ compared with iM0φ.

b. GO and KEGG analysis of the genes or pathways that were upregulated or enriched in iM2φ compared with iM0φ.

**Supplementary Fig. 9: Characterization of iM1φ and iM2φ cells polarized from hPSC-derived macrophages**

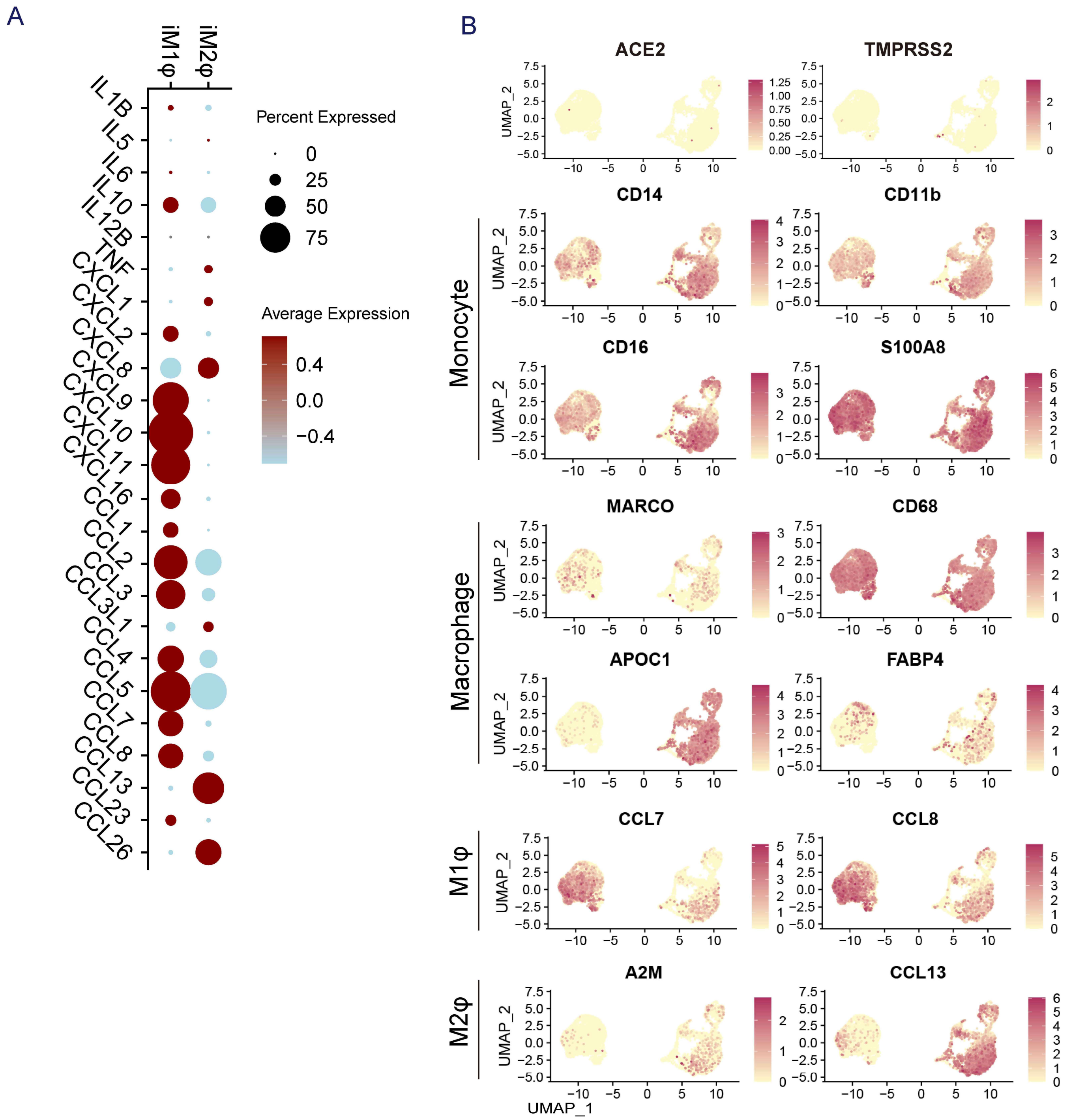


**Supplementary Fig. 9: Characterization of iM1φ and iM2φ cells polarized from hPSC-derived macrophages.**

scRNA seq was performed on the co-cultures (iLung and iM1φ co-culture; iLung and iM2φ co-culture), (See Figure S8 on iLung)

a. Dot plot presenting the cytokine and chemokine related genes in iM1φs and iM2φs. Relative expression of each gene ranges from low (light blue) to high (red) as indicated.

b. ACE2, TMPRSS2, putative cell-fate related markers, and inflammatory factors differentially expressed in the cluster of iM1φ and iM2φ in UMAPs. Relative expression of each marker gene ranges from low (light yellow) to high (red) as indicated. Individual cell positive for each marker are donated by red dots.

## Supplementary Fig. 10: Characterization of the co-culture of lung cells and macrophages derived from hPSCs

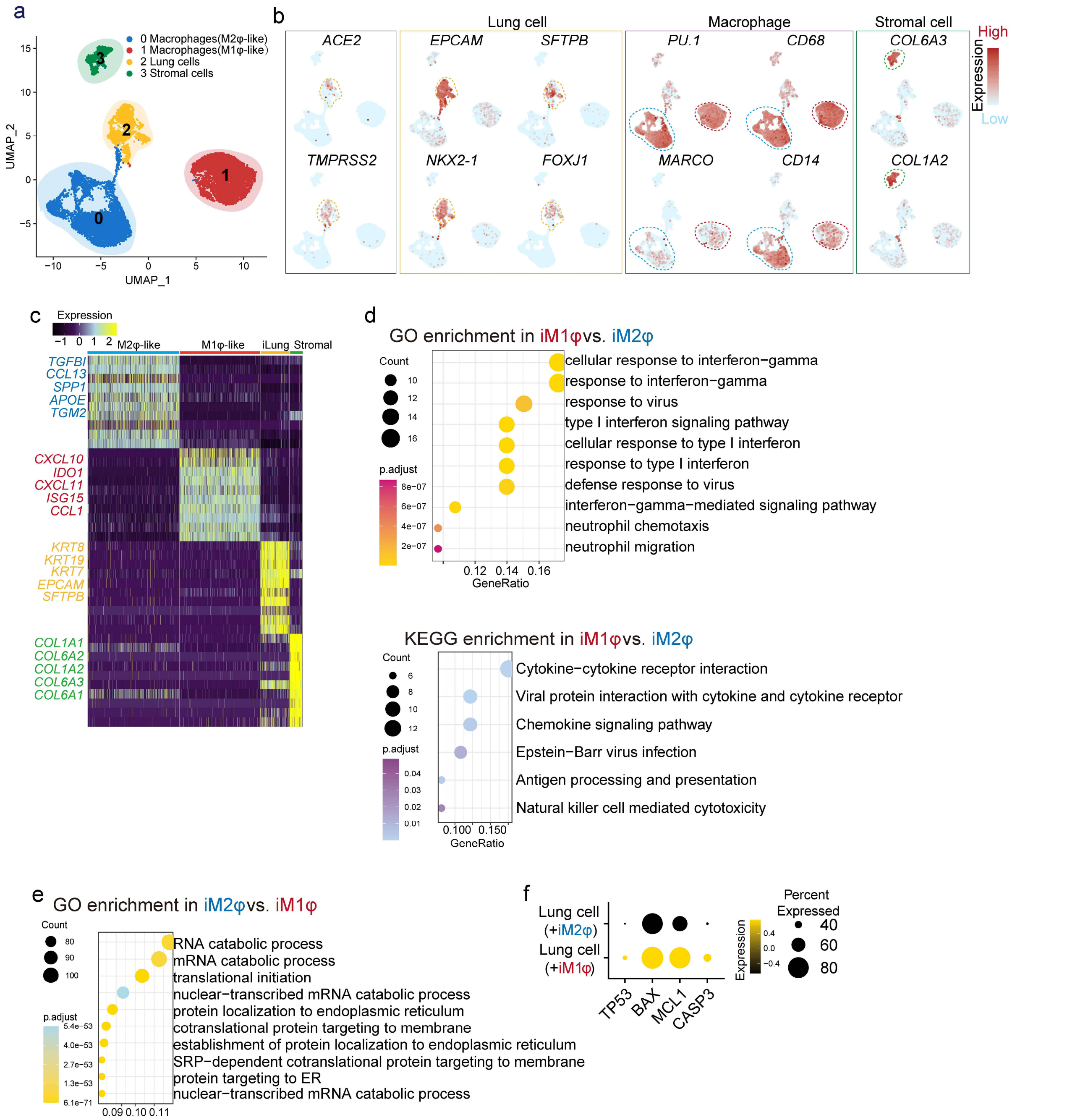


**Supplementary Fig. 10: Characterization of the co-culture of lung cells and macrophages derived from hPSCs**

a. UMAP representing scRNA seq of the two co-cultures (iLung and iM1φ co-culture; iLung and iM2φ co-culture). Colored and annotated with cluster 0-3 representing iM2φ, iM1φ, lung epithelial cells and stromal cells.
b. ACE2, TMPRSS2, as well as putative cell-fate related markers differentially expressed in each cluster in UMAPs. Relative expression of each marker gene ranges from low (light blue) to high (pink) as indicated. Individual cells positive for each marker are donated by red dots. The main population of ACE2 or TMPRSS2 positive populations are circled in dotted line.
c. Heatmap examination of iM2φ, iM1φ, lung epithelial cells and stromal cells for top differentially expressed genes.
d. Gene set enrichment analysis by GO (Gene Ontology) and KEGG (Kyoto Encyclopedia of Genes and Genomes) in iM1φs, versus iM2φs.
e. GO Gene set enrichment analysis in iM2φs, versus iM1φs.

f. Dot plot of cell-death related genes in iLung cells co-cultured with iM1φs or iM2φs. Relative expression levels of each gene range from low (black) to high (yellow) as indicated. Percentages of cells with differential gene expression are indicated by the size of circles.

**Supplementary Fig. 11: ACE2 expression in hPSC-lung cells and iMACs**

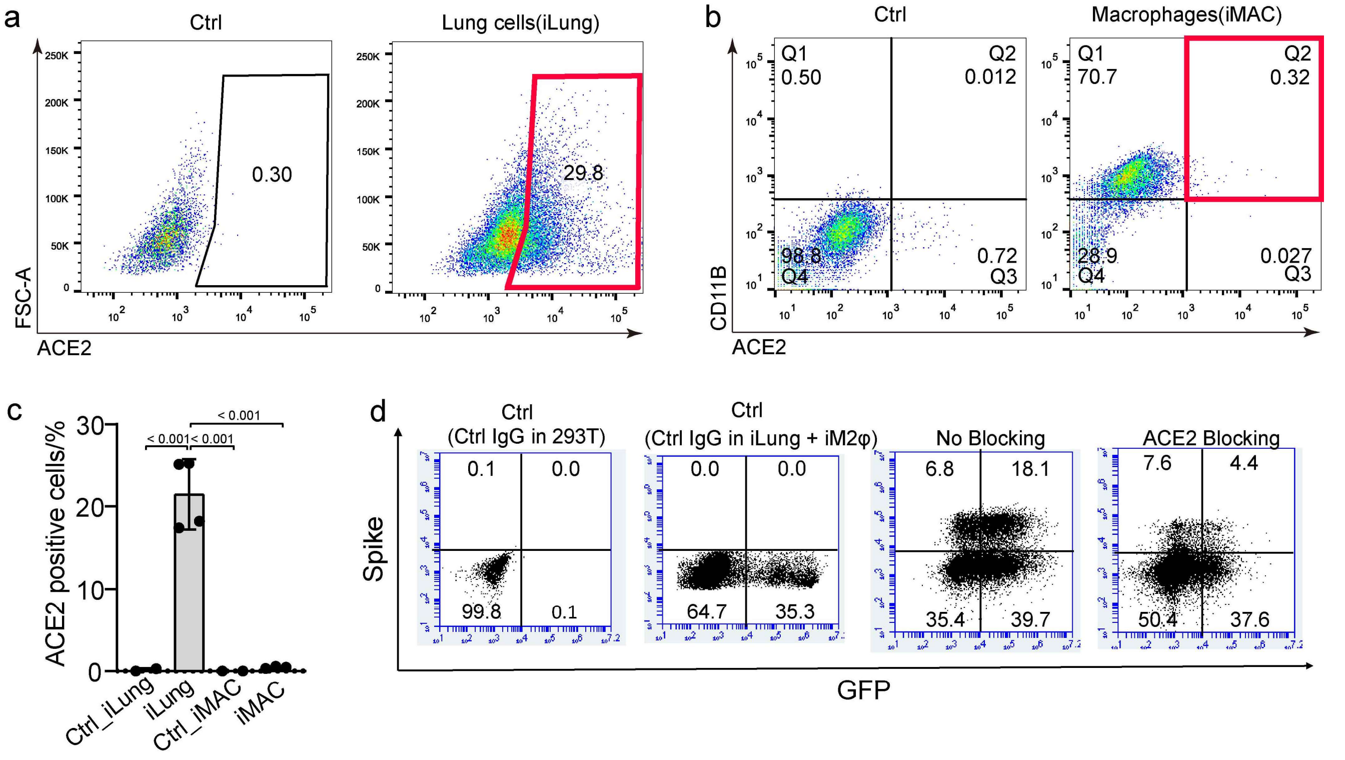


**Supplementary Fig. 11: ACE2 expression in hPSC-lung cells and iMACs**

a. Flow cytometry characterized ACE2 exprssion in hPSC-derived lung cells.

b. Flow cytometry characterized ACE2 exprssion in hPSC-derived macrophages. CD11B+ gated cells represent the iMACs population.

c. Bar graph represents the quantification of ACE2 expression in hPSC-lung cells and iMACs. n = 3 independent experiments, data are presented as mean values ± standard deviation. Statistically significant differences are calculated using an unpaired two-tailed unpaired Student's t-test.

d. Representative FACS images for Figure 5F.

**Supplementary Fig. 12: the dynamics of SARS-CoV-2 in different types of macrophages**

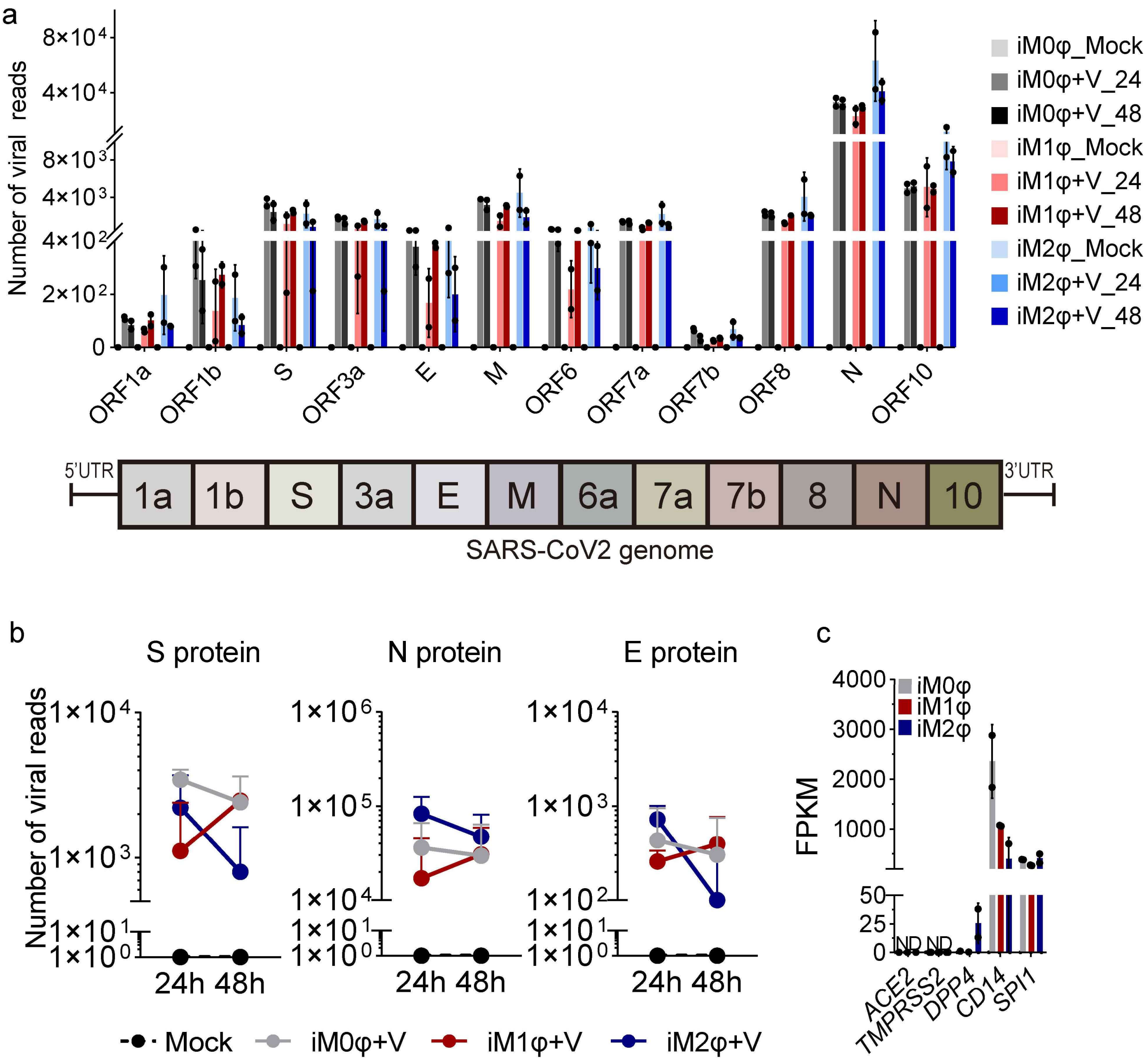


**Supplementary Fig. 12: the dynamics of SARS-CoV-2 in different types of macrophages.**

a. Viral reads coverage along the SARS-CoV-2 genome for macrophages were assessed using RNA-seq. Macrophages infected at 24hpi and 48hpi were compared with mock-treated cells. A model of SARS-CoV-2 genome is shown below. n = 2 independent experiments, data are presented as mean values ± standard deviation.

b. The changes of viral spike(S), nucleocapsid(N) and envelop(E) protein in macrophages at 24 and 48 hpi. n = 3 independent experiments, data are presented as mean values ± standard deviation.

c. The expression level of putative receptors for SARS-CoV-2 (ACE2/TMPRSS2), receptor for MERS(DPP4) and pan markers for macrophages (CD14 and SPI1). n = 2 independent experiments, data are presented as mean values ± standard deviation.

**Supplementary Fig. 13: Characterization of Macrophages following SARS-CoV-2 infection**

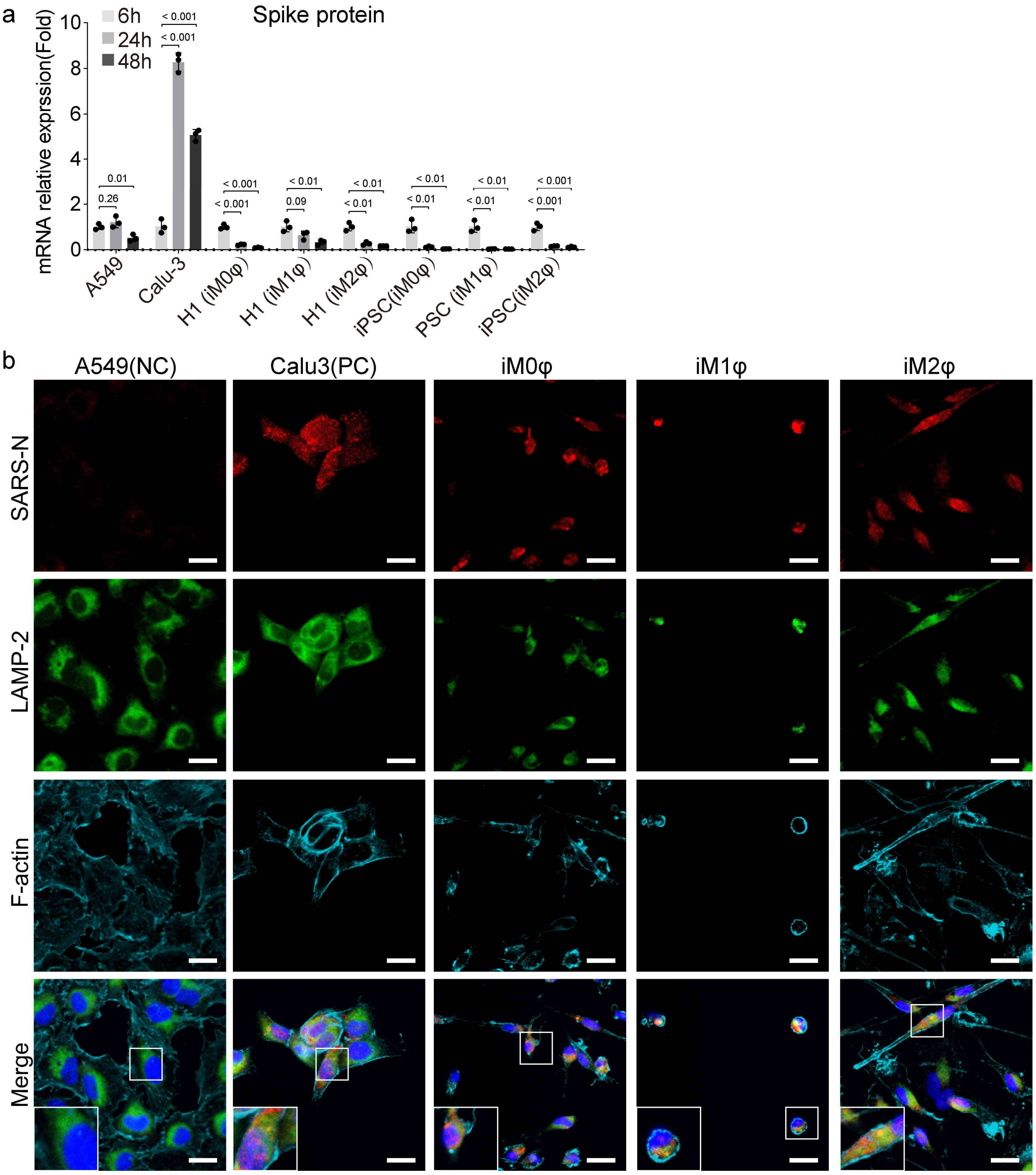


**Supplementary Fig. 13: Characterization of Macrophages following SARS-CoV-2 infection**

a. Bar graph depicts RT-qPCR analysis of mRNA expression of SARS-CoV-2 spike protein in control cells and iMACs after SARS-CoV-2 infection at 6hpi ,24hpi, and 48 hpi. n = 3 independent experiments. Results are indicated as mean ± standard deviation. Statistically significant differences are calculated using an unpaired two-tailed unpaired Student's t-test.

b. IF staining of A549(negative control), Calu3(positive control), and iMφs (iM0φ/iM1φ/iM2φ) against SARS-N and lysosome marker, LAMP-2. Cytoskeleton was labeled by F-actin. Scale bars = 10μm.

**Supplementary Fig. 14: the immune response of macrophages after SARS-CoV-2 infection**

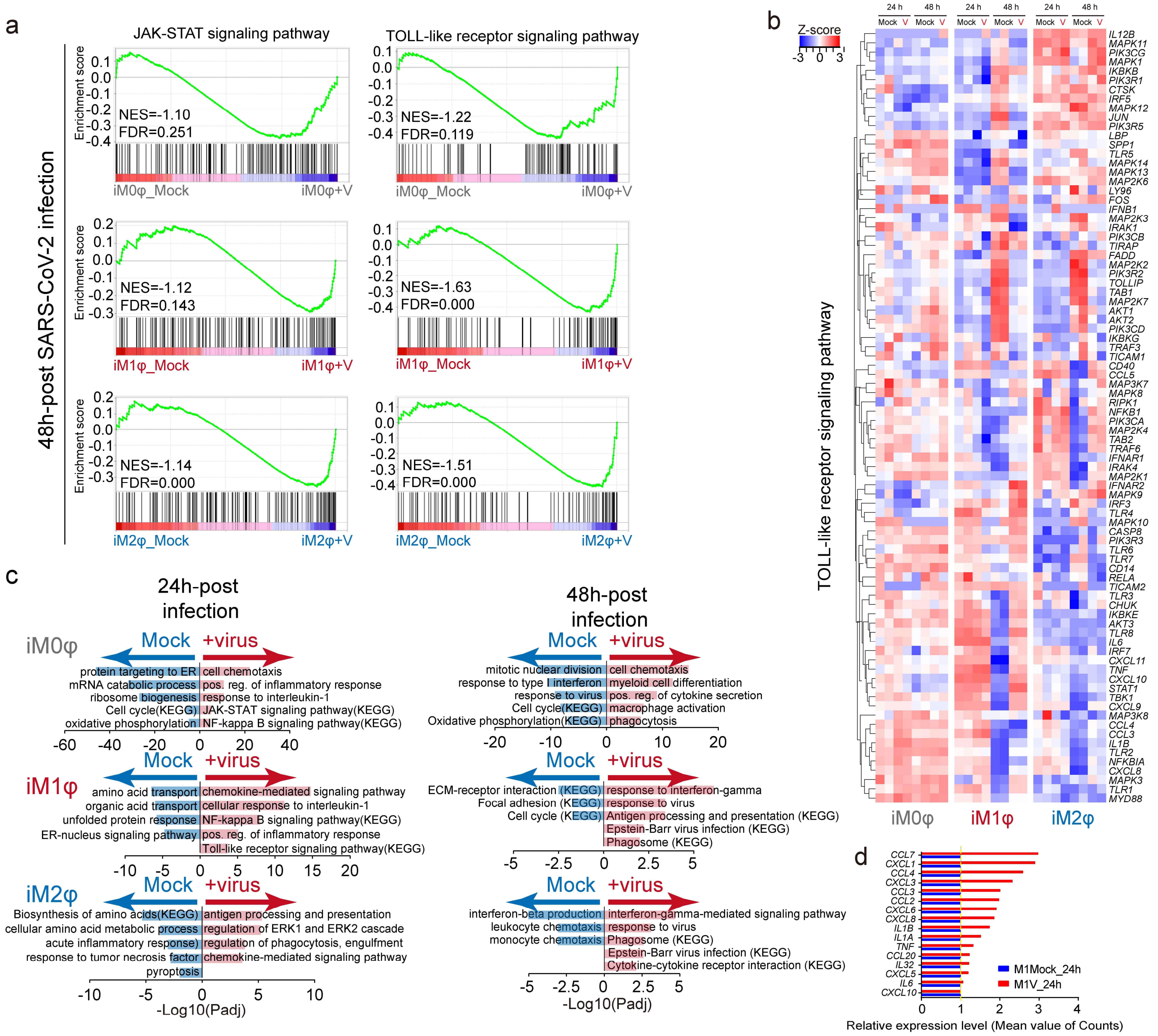


**Supplementary Fig. 14: the immune response of macrophages after SARS-CoV-2 infection.**

a. GSEA of JAK-STAT and Toll-like signaling pathways in iM0φs, iM1φs, and iM2φs 48 hours following SARS-CoV-2 infection

b. Heatmap of the genes related to Toll-like signaling pathways in iM0φs, iM1φs, and iM2φs 24 and 48hours following SARS-CoV-2 infection.

c. GO and KEGG analysis of the activation of immune-relevant pathways by comparing iM0φs, iM1φs, and iM2φs infected with SARS-CoV-2 at 24 and 48hpi with the mock.

d. The relative expression level of inflammatory factors in iM1φ before and after infection with SARS-CoV-2 virus. The count values representing gene expression level was derived from the bulk RNA-seq and the average was calculated.
