# Supplementary material for: Differential effects of macrophage subtypes on SARS-CoV-2 infection in a human pluripotent stem cell-derived model
Source: Nat Commun. 2022 Apr 19;13:2028. doi: 10.1038/s41467-022-29731-5 (PMC9018716; doi:10.1038/s41467-022-29731-5)
Supplement: Supplementary file 2 — Reporting Summary [file 41467_2022_29731_MOESM2_ESM.pdf]

nature research

Corresponding author(s): Huanhuan Joyce Chen, Qizhou Lian

Last updated by author(s): Feb 8, 2022

Reporting Summary

Nature Research wishes to improve the reproducibility of the work that we publish. This form provides structure for consistency and transparency in reporting. For further information on Nature Research policies, see our Editorial Policies and the Editorial Policy Checklist.

Statistics

For all statistical analyses, confirm that the following items are present in the figure legend, table legend, main text, or Methods section.

n/a	Confirmed	
[ ]	[x]	The exact sample size (*n*) for each experimental group/condition, given as a discrete number and unit of measurement
[ ]	[x]	A statement on whether measurements were taken from distinct samples or whether the same sample was measured repeatedly
[ ]	[x]	The statistical test(s) used AND whether they are one- or two-sided<br>*Only common tests should be described solely by name; describe more complex techniques in the Methods section.*
[ ]	[x]	A description of all covariates tested
[ ]	[x]	A description of any assumptions or corrections, such as tests of normality and adjustment for multiple comparisons
[ ]	[x]	A full description of the statistical parameters including central tendency (e.g. means) or other basic estimates (e.g. regression coefficient) AND variation (e.g. standard deviation) or associated estimates of uncertainty (e.g. confidence intervals)
[ ]	[x]	For null hypothesis testing, the test statistic (e.g. *F*, *t*, *r*) with confidence intervals, effect sizes, degrees of freedom and *P* value noted<br>*Give P values as exact values whenever suitable.*
[x]	[ ]	For Bayesian analysis, information on the choice of priors and Markov chain Monte Carlo settings
[x]	[ ]	For hierarchical and complex designs, identification of the appropriate level for tests and full reporting of outcomes
[x]	[ ]	Estimates of effect sizes (e.g. Cohen's *d*, Pearson's *r*), indicating how they were calculated

Our web collection on statistics for biologists contains articles on many of the points above.

Software and code

Policy information about availability of computer code

Data collection	Single-cell capture, reverse transcription, cell lysis, and library preparation was performed using the Single Cell 3′ version 3 kit and chip according to the manufacturer's protocol (10x Genomics).Sequencing was performed on NovaSeq6000 with setting 28 for read 1 and 91 for read 2. The sequencing data were primarily analyzed by CellRanger pipeline v3.0.2 (10x Genomics).<br>For bulk RNA-seq, messenger RNA was prepared using TruSeq Stranded mRNA Sample Library Preparation kit (Illumina), according to the manufacturer's instructions. The normalized libraries were pooled and sequenced on Illumina Novaseq 6000 sequencer with pair-end 50 cycles.The sequencing libraries sequenced with paired-end 50 bps on NovaSeq6000 sequencer. (See details in Method section and sources data)
Data analysis	The following softwares were used for data analysis (See details in Method section and sources data):<br>Cell Ranger(v3.0.2) https://support.10xgenomics.com/single-cell-gene-expression/software/overview/welcome<br>scran R package(v1.14.1) https://bioconductor.org/packages/release/bioc/html/scran.html<br>Rstudio (v1.1.4) https://rstudio.com/<br>Seurat R package(v3.1.4) https://satijalab.org/seurat/<br>Cutadapt(v1.18) https://cutadapt.readthedocs.io/en/v1.18/<br>STAR(v2.5.2) https://github.com/alexdobin/STAR<br>Cufflinks(v2.1.1) http://cole-trapnell-lab.github.io/cufflinks/<br>HTSeq (v0.11.2) https://htseq.readthedocs.io/en/master/<br>DESeq2(v1.22.2) http://bioconductor.org/packages/release/bioc/html/DESeq2.html<br>R ggplot2 package(3.3.2) https://cran.r-project.org/web/packages/ggplot2/index.html<br>R clusterProfiler package(v3.10.1) https://bioconductor.org/packages/release/bioc/html/clusterProfiler.html<br>GSEA software (v4.0.3) https://www.gsea-msigdb.org/gsea/index.jsp<br>Adobe illustrator CC2017 https://www.adobe.com/products/illustrator.html

Graphpad Prism 8.0 https://www.graphpad.com/scientific-software/prism/
FlowJo v x.0.7 https://www.flowjo.com/
ToppCell Atlas https://toppgene.cchmc.org/

For manuscripts utilizing custom algorithms or software that are central to the research but not yet described in published literature, software must be made available to editors and reviewers. We strongly encourage code deposition in a community repository (e.g. GitHub). See the Nature Research guidelines for submitting code & software for further information.

Data

Policy information about availability of data

All manuscripts must include a data availability statement. This statement should provide the following information, where applicable:

- Accession codes, unique identifiers, or web links for publicly available datasets
- A list of figures that have associated raw data
- A description of any restrictions on data availability

The scRNA-seq data are available from the GEO repository database with accession number GSE162996 (hPSC-derived lung cells, co-culture of macrophage and lung cells derived from hPSC).
https://www.ncbi.nlm.nih.gov/geo/query/acc.cgi?acc=GSE162996
Bulk RNA-seq data are accessible through GEO Series accession number GSE160631 (macrophages derived from hPSC, macrophages derived from hPSC in SARS-CoV-2 infection).
https://www.ncbi.nlm.nih.gov/geo/query/acc.cgi?acc=GSE160631
(See details in Method section and sources data)

Field-specific reporting

Please select the one below that is the best fit for your research. If you are not sure, read the appropriate sections before making your selection.

☒ Life sciences ☐ Behavioural & social sciences ☐ Ecological, evolutionary & environmental sciences

For a reference copy of the document with all sections, see nature.com/documents/nr-reporting-summary-flat.pdf

Life sciences study design

All studies must disclose on these points even when the disclosure is negative.

Sample size	Sample sizes were determined to be adequate for minimal n required for statistical tests. Sample sizes for all figures and tables were estimated based on our previous studies:<br>Chen, H.J. et al. Generation of pulmonary neuroendocrine cells and SCLC-like tumors from human embryonic stem cells. J. Exp. Med. 216, 674-687 (2019).<br>Huang, S.X. et al. Efficient generation of lung and airway epithelial cells from human pluripotent stem cells. Nat. Biotechnol. 32, 84 (2014).<br>Han, Y. et al. Identification of SARS-CoV-2 inhibitors using lung and colonic organoids. Nature 589, 270-275 (2021).<br>Huang, S.X. et al. The in vitro generation of lung and airway progenitor cells from human pluripotent stem cells. Nat. Protoc. 10, 413-425 (2015).
Data exclusions	For each set of experiments, samples were prepared for all experimental arms at the same time. No data were excluded from the study design. All statistical tests are 2-sided. No adjustments were made for multiple comparisons.
Replication	All experiment were replicated at least three times and all attempts at replication are successful.
Randomization	The cells or specimen were randomly separated into different groups for different treatments.
Blinding	The relevant investigators (KZ, ZZ, FD and LG) were blinded to experimental allocations among different experimental arms for all experiments.

Reporting for specific materials, systems and methods

We require information from authors about some types of materials, experimental systems and methods used in many studies. Here, indicate whether each material, system or method listed is relevant to your study. If you are not sure if a list item applies to your research, read the appropriate section before selecting a response.

Materials & experimental systems

n/a	Involved in the study	
☐	☒	Antibodies
☐	☒	Eukaryotic cell lines
☒	☐	Palaeontology and archaeology
☒	☐	Animals and other organisms
☐	☒	Human research participants
☒	☐	Clinical data
☒	☐	Dual use research of concern

Methods

n/a	Involved in the study	
☒	☐	ChIP-seq
☐	☒	Flow cytometry
☒	☐	MRI-based neuroimaging

Antibodies

Antibodies used

Mouse monoclonal anti-CD68 eBioscience #14-0688-82
Rabbit polyclonal anti-CD80 BOSTER #A00196-1
CD80-PE, human miltenyi Biotec #130-117-683
CD206-PE, human miltenyi Biotec #130-095-220
Rabbit monoclonal anti-CD163 Abcam #ab182422
Rabbit polyclonal anti-IL6 Affinity #DF6087
Goat polyclonal anti-ACE2 R&D system #AF933
PE-conjugated CD43 eBioscience #eBio84-3C1
APC-conjugated CD34 BD Biosciences clone 581
PE-conjugated CD68 Biolegend clone Y1/82A
APC-conjugated CD11b Biolegend clone ICRF44
FITC-conjugated CD14 Biolegend clone HCD14
Anti-NKX2.1 Antibody Seven Hills Bioreagents #WRAB-1231
Anti-FOXA2 Antibody Santa Cruz #sc-6554
Anti-SOX2 Antibody Santa Cruz #sc-17320
Anti-SP-B Antibody Seven Hills Bioreagents #WRAB-48604
Anti-Pro-SP-C Antibody Seven Hills Bioreagents #WRAB-9337
Anti-FOXJ1 Antibody Sigma-Aldrich #HPA005714-1
Anti-SARS-CoV-2 Nucleocapsid Antibody R&D system #MAB10474
Mouse Anti-SARS-CoV-Spike antibody Provided by Dr. Tom Moran #2B3E5
Firefly luciferase Monoclonal Antibody (CS 17) Thermo Fisher Scientific #35-6700
Recombinant Anti-Firefly Luciferase antibody Abcam #ab185924
Donkey anti-Mouse IgG (H+L) Highly Cross-Adsorbed Secondary Antibody, Alexa Fluor 488 Thermo Fisher Scientific #A-21202
Alexa Fluor 488 AffiniPure Donkey Anti-Guinea Pig IgG (H+L) Jackson Immunoresearch Labs #706-545-148
Donkey anti-Mouse IgG (H+L) Highly Cross-Adsorbed Secondary Antibody, Alexa Fluor 594 Thermo Fisher Scientific #A-21203
Donkey anti-Rabbit IgG (H+L) Secondary Antibody, Alexa Fluor 594 conjugate Thermo Fisher Scientific #A-21207
Donkey anti-Rabbit IgG (H+L) Secondary Antibody, Alexa Fluor 647 conjugate Thermo Fisher Scientific #A-31573onkey anti-Mouse IgG (H+L) Secondary Antibody, Alexa Fluor 647 Thermo Fisher Scientific #A-31571
Donkey anti-Goat IgG (H+L) Cross-Adsorbed Secondary Antibody, Alexa Fluor 647 Thermo Fisher Scientific #A-21447
Donkey anti-Chicken IgG (H+L) Cross-Adsorbed Secondary Antibody, Alexa Fluor 488 Jackson Immunoresearch Labs #703-545-155
Donkey anti-Sheep IgG (H+L) Cross-Adsorbed Secondary Antibody, Alexa Fluor 647 Thermo Fisher Scientific #A-21448

Validation

1. Anti-CD68 (eBioscience, 14-0688-82) was verified by Cell treatment to ensure that the antibody binds to the antigen stated. Used in 19 publications based on manufacturer website.
2. Anti-CD68 (Abcam, ab213363) have been verified in various human cell lines and tissues, including Kupffer cells, U937, THP-1, spleen, tonsil, fetal liver, and fetal spleen. Used in 29 publications based on manufacturer website
3. Boster Bio Anti-CD80 Antibody Picoband™ catalog # A00196-1. Tested in ELISA, IHC applications. This antibody reacts with Human, Rat. Cited in 2 publication(s).
4. Anti-CD163 (Abcam, ab156769) have been verified in human liver tissue, skeletal muscle, COS-7, and HEK-293T. Used in 18 publications based on manufacturer website.
5. Anti-CD163 (Abcam, ab182422), produced recombinantly (animal-free) for high batch-to-batch consistency and long-term security of supply. Suitable for: Flow Cyt, IHC-P, WB, IHC-Fr. Reacts with: Mouse, Rat, Human. This antibody has been used in mouse spleen, liver; human PBMC cells, human fetal liver, tonsil, spleen placenta, first trimester placenta, and breast carcinoma; Rat liver, muscle, and achilles tissues. Used in 171 publications based on manufacturer website.
6. Anti-CD80-PE (miltenyi Biotec, 130-117-683) is verified in peritoneal macrophages from C57BL/6 miceusing flow cytometry. Used in 2 publications based on manufacturer website
7. Anti-CD206-PE (miltenyi Biotec, 130-095-220) has been used in 4 publications based on manufacturer website
8. Anti-CD206 (Abcam, ab64693) has been verified in rat liver and lung; mouse lung; human lung and MOLT-3 cell line. Used in 427 publications based on manufacturer website
9. Anti-FABP4 (Abcam, ab92501) has been verified in mouse brown adipose, heart, kidney, lung and embryonic fibroblast; human adipose, breast and fetal heart; rat adipose. Used in 60 publications based on manufacturer website
10. Anti-HLA-DR (eBioscience, 14-9956-80) was verified by Relative expression to ensure that the antibody binds to the antigen

stated. Used in 26 publications based on manufacturer website.
11. Anti-IL6 (Affinity, DF6087) was verified using western blot in various tissues, including rat spleen, heart, and muscle, human breast cancer. Used in 65 publications based on manufacturer website
12. Anti-IL-32 (Abcam, ab37158) was verified in human spleen. Used in 14 publications based on manufacturer website.
13. Anti-CCL2 (Abcam, ab9858) has been verified using ELISA with recombinant human MCP-1. Used in 3 publications based on manufacturer website.
14. Anti-IL-1 beta (Abcam, ab254360) has been verified in wild-type THP-1 cells and IL1B knockout THP-1 cells. Used in 3 publications based on manufacturer website.
15. Anti-ACE2 (R&D systems, AF933), Detects human ACE-2 in direct ELISAs. Detects human, mouse, and rat ACE-2 in Western blots. Detects Hamster ACE-2 in immunohistochemistry. In direct ELISAs and Western blots, less than 1% cross-reactivity with recombinant human ACE is observed. Used in 63 publications based on manufacturer website
16. Anti-CD68-PE (Biolegend, 333807) has been used in 2 publications based on manufacturer website.
17. Anti-CD11b, APC-conjugated (Biolegend, 301309) antibody inhibits heterotypic adhesion of granulocytes in response to fMLP. Additional reported applications (for the relevant formats) include: immunohistochemical staining of acetone-fixed frozen tissue sections, immunofluorescence microscopy5, stimulation of monocytes3, blocking of heterotypic PMN aggregation8, and blocking of granulocyte activation12. This clone was tested in-house and does not work on formalin fixed paraffin embedded (FFPE) tissue. Used in 38 publications based on manufacturer website
18. Anti-CD14, FITC-conjugated (Biolegend, 325603) can be used in human, used in 38 publications based on manufacturer website
19. Anti-NKX2.1 (Seven Hills Bioreagents, WRAB-1231) can recognize human & mouse NKX2.1, other species not tested. Used in 4 publications based on manufacturer website
20. Anti-FOXA2 (Santa Cruz, sc-6554) is recommended for detection of FOXA2 of mouse, rat and human origin by western Blotting, immunoprecipitation, immunofluorescence, immunohistochemistry, and solid phase ELISA. Used in 66 publications based on manufacturer website
21. Anti-SOX2 (Santa Cruz, sc-17320) antibody is recommended for detection of Sox-2 of mouse, rat and human origin by Western Blotting, immunoprecipitation, immunofluorescence, immunohistochemistry, and solid phase ELISA. Used in 119 publications based on manufacturer website
22. Anti-SP-B (Seven Hills Bioreagents, WRAB-48604) antibody can recognize human, mouse, cow & sheep SP-B, other species not tested. Used in 3 publications based on manufacturer website
23. Anti-Pro-SP-C (Seven Hills Bioreagents, WRAB-9337) can recognize human & mouse pro SP-C, other species not tested. Used in 7 publications based on manufacturer website
24. Anti-FOXJ1 (Sigma-Aldrich, HPA005714-1) antibody produced in rabbit, a Prestige Antibody, is developed and validated by the Human Protein Atlas (HPA) project (www.proteinatlas.org). Each antibody is tested by immunohistochemistry against hundreds of normal and disease tissues. These images can be viewed on the Human Protein Atlas (HPA) site by clicking on the Image Gallery link. The antibodies are also tested using immunofluorescence and western blotting. Used in 15 publications based on manufacturer website
25. Anti-SARS-CoV-2 Nucleocapsid Antibody (R&D systems, MAB10474), detects SARS-CoV-2 Nucleocapsid in direct ELISAs and Western blots. No cross-reactivity with MERS Nucleocapsid is observed in Western blots. Used in 7 publications based on manufacturer website
26. SARS-CoV/SARS-CoV-2 Nucleocapsid Antibody (Sino Biological, 40143-MM05) has been validated and can be used in WB, ELISA, IHC-P, and FCM. Used in 18 publications based on manufacturer website.
27. Anti-LAMP2 (Santa Cruz, sc-5571) is recommended for detection of LAMP-2 of human and, toa lesser extent, mouse and rat origin by Western Blotting, immunoprecipitation, immunofluorescence, immunohistochemistry, and solid phase ELISA. Used in 10 publications based on manufacturer website.

Eukaryotic cell lines

Policy information about cell lines

Cell line source(s)	RUES2 and H1 hESC lines were purchased from WiCell. HEK293T(CRL-11268), Vero E6(CRL-1586) and THP-1(TIB-202) cell lines were purchased from ATCC. Mouse embryonic fibroblasts(GSC-6001G) were purchased from GSC-6001G.<br>For Vero cell lines, no Vero cell line was used in the studies reported in the manuscript, though we discussed the application of Vero cell in COVID-19 research in the discussion section.
Authentication	Cell lines were authenticated by DNA fingerprinting analysis.
Mycoplasma contamination	All cell lines were confirmed to be mycoplasma free. The cells lines purchased from ATCC which certified the lots to be free of Mycoplasma contamination.
Commonly misidentified lines (See ICLAC register)	No misidentified cell lines were used in this study

Human research participants

Policy information about studies involving human research participants

Population characteristics

The paraffin-embedded lung tissues were acquired from the department of pathology in the third people's hospital of Shenzhen, China. They reported the pathological changes of lungs in critical COVID-19 infection (a 66 year-old male, a 62 year-old female, and a 31 year-old male; all patients are Asian Chinese). The diagnosis of COVID-19 pneumonia was based on the "Coronavirus Pneumonia Prevention and Control Plan" (7th edition) newly issued by the National Health Commission, China 48. Nasopharyngeal swabs were collected and COVID-19 was detected by real-time polymerase chain reaction. Infection was defined as at least two positive test results. The COVID patients developed respiratory failure and septic shock during the treatment. Informed consent was obtained from the patients and families.

For non-COVID-19 paraffin-embedded lung tissues, the samples were obtained from two patients undergoing elective surgery who were diagnosed with chronic bronchitis (a 47-year-old male, Asian Chinese) or lung cancer before chemotherapies (a 59-year-old female, Asian Chinese) respectively. Diseased lung tissues were removed as part of routine clinical care but surplus for routine diagnostic requirements. This study was approved by the Institutional Review Board of the third People's hospital of Shenzhen (2021-0089). Surgical informed consent was obtained from patients or guardians before the sample collection. (See more details in Method section)

Recruitment

The human tissues on both COVID and control groups were obtained with randomization, including both male and female Asian/Chinese participants that are comparable in terms of observable and unobservable characteristics.

Ethics oversight

The study was performed on paraffin-embedded lung tissues of COVID-19 patients and non-COVID-19 controls, were acquired from the department of pathology in the 3rd people's hospital of Shenzhen, China. All research activities with human lung specimens of COVID-19 patients and non-COVID-19 controls were implemented under the protocol approved by the institutional research ethics committee of 3rd hospital of Shenzhen (No 2021-0089). Informed consent was obtained from the patients and families. Participants were not compensated.
All embryonic stem cell studies were approved by the Institutional Review Board (IRB, IRB20-0598) at the University of Chicago, or by the Tri-Institutional ESCRO committee (Weill Cornell Medicine, Memorial Sloan Kettering Cancer Center, and Rockefeller University). (Details are also described in the Method section)

Note that full information on the approval of the study protocol must also be provided in the manuscript.

Flow Cytometry

Plots

Confirm that:

- [x] The axis labels state the marker and fluorochrome used (e.g. CD4-FITC).
- [x] The axis scales are clearly visible. Include numbers along axes only for bottom left plot of group (a 'group' is an analysis of identical markers).
- [x] All plots are contour plots with outliers or pseudocolor plots.
- [x] A numerical value for number of cells or percentage (with statistics) is provided.

Methodology

Sample preparation

For extracellular staining, basically cells were resuspended in a FACS buffer (PBS with 0.1 % BSA and 2.5 mM EDTA) and incubated with antibodies for 30 mins at 4°C, followed with washed and suspended in 0.1% BSA/PBS buffer. For intracellular staining, cells were fixed with 2% paraformaldehyde for 20 mins at room temperature. For permeabilization, cells were resuspended in 0.2% Triton-X100 in PBS for 10 mins, followed with washed and suspended in 0.1% BSA/PBS buffer, the following procedures were the same as the extracellular staining. Negative controls stained with control IgG instead of primary antibodies were always performed with sample measurements. Flow cytometry machine of BD BD LSR Fortessa and software of FlowJo (Version x.0.7) were mainly used to collect and analyze the flow cytometry data. (See details described in the Method section)

Instrument

BD LSR Fortessa

Software

FlowJo (Version x.0.7)

Cell population abundance

Positive cells were above 98% of the total cells in the post-sorting fractions, as the sorted populations were re-analyze by running through the FACS for the second analysis.

Gating strategy

All recorded events were gated according to FSC and SSC; single cells were selected using FSC-H vs. FSC-W.Negative controls stained with control IgG . The gating strategies have been presented alone with each FACS figure (Figure S6, and Figure S11)

- [x] Tick this box to confirm that a figure exemplifying the gating strategy is provided in the Supplementary Information.
